# Towards new sources of resistance to the currant-lettuce aphid (*Nasonovia ribisnigri*)

**DOI:** 10.1007/s11032-016-0606-4

**Published:** 2017-01-03

**Authors:** Peter G. Walley, Gemma Hough, Jonathan D. Moore, John Carder, Marian Elliott, Andrew Mead, Julie Jones, Graham Teakle, Guy Barker, Vicky Buchanan-Wollaston, Paul Hand, David Pink, Rosemary Collier

**Affiliations:** 1 Functional and Comparative Genomics, Institute of Integrative Biology, University of Liverpool, Liverpool, L69 7ZB UK; 2 Syngenta, Jealott’s Hill International Research Centre, Bracknell, Berkshire RG42 6EY UK; 3 Earlham Institute, Norwich Research Park, Norwich, NR4 7UH UK; 4 School of Life Sciences, Warwick Crop Centre, The University of Warwick, Wellesbourne, Warwick, CV35 9EF UK; 5 Rothamsted Research, West Common, Harpenden, Hertfordshire AL5 2JQ UK; 6 Harper Adams University College, Newport, Shropshire TF10 8NB UK

**Keywords:** Lettuce, Diversity, SNP, Aphid, Association, Resistance

## Abstract

Domesticated lettuce varieties encompass much morphological variation across a range of crop type groups, with large collections of cultivars and landrace accessions maintained in genebanks. Additional variation not captured during domestication, present in ancestral wild relatives, represents a potentially rich source of alleles that can deliver to sustainable crop production. However, these large collections are difficult and costly to screen for many agronomically important traits. In this paper, we describe the generation of a diversity collection of 96 lettuce and wild species accessions that are amenable to routine phenotypic analysis and their genotypic characterization with a panel of 682 newly developed expressed sequence tag (EST)-linked KASP™ single nucleotide polymorphism (SNP) markers that are anchored to the draft *Lactuca sativa* genome assembly. To exemplify the utility of these resources, we screened the collection for putative sources of resistance to currant-lettuce aphid (*Nasonovia ribisnigri*) and carried out association analyses to look for potential SNPs linked to resistance.

**Electronic supplementary material:**

The online version of this article (doi:10.1007/s11032-016-0606-4) contains supplementary material, which is available to authorized users.

## Introduction

Lettuce (*Lactuca sativa* L.) is a high-value horticultural crop in many countries, e.g. UK lettuce production/imports had an estimated farm gate value of £266 million in 2011 (Defra 2012) to which significant value is added through minimal processing, into ‘ready to eat’ salad packs (Altunkaya and Gokmen 2008). This growing sector is linked to the perception of lettuce being a healthy food option (Anderson et al. 2007). Mintel (2007) estimated the retail value of UK processed salads to be nearly £800 million; more recently, global lettuce and chicory production was estimated at over 24.8 million tonnes for the calendar year 2013 (FAOSTAT 2016), further emphasizing the economic importance of this crop.

Producers of high-value salad packs require high-quality raw material free from blemishes and ‘foreign’ bodies including insects. The currant-lettuce aphid *Nasonovia ribisnigri* (Mosley) (Hemiptera, Aphididae) is the most significant pest infesting lettuce in northern Europe (Collier et al. 1999; Reinink and Dieleman 1993). Its presence at harvest makes heads and salad packs unmarketable with significant financial losses for growers (Parker et al. 2002). Ensuring aphid-free lettuce is a particular problem for growers due to the aphids’ preference to feed at the centre of lettuce heads where they are difficult to control with foliar insecticides (Aarts et al. 1999). Furthermore, strains of *N. ribisnigri* have been found with varying levels of resistance to pirimicarb, pyrethroid and organophosphate insecticides (Barber et al. 1999; Barber et al. 2002; Kift et al. 2004; Rufingier et al. 1999). Until recently, the most effective control method for *N. ribisnigri* was the use of resistant cultivars of lettuce.

Resistance was identified initially in several accessions of the related wild species *L. virosa*, a member of the secondary gene pool for lettuce (Eenink et al. 1982b). The resistance provided nearly complete control of the prevalent strain of *N. ribisnigri*, now referred to as biotype Nr:0. Genetic analyses revealed that resistance is controlled by one dominant gene, known as *Nr* (Eenink et al. 1982a, b; Eenink and Dieleman 1983). Interspecific crosses between the *L. virosa* accessions and lettuce were not successful, so the wild species *L* *actuca serriola* was used as a bridging species to introgress the resistance into lettuce (Eenink et al. 1982b). The resultant pre-breeding lines were released to breeding companies who have since incorporated *Nr* into a large proportion of modern cultivars (van der Arend 2003). These resistant cultivars are grown widely, but the selection pressure induced by reliance on a single resistance gene has resulted in a new currant-lettuce aphid biotype (*N. ribisnigri* biotype Nr:1) that is able to thrive on ‘resistant’ plants possessing *Nr* (Smilde et al. 2009). The identification of new mechanisms of resistance is therefore required urgently.

The screening of large numbers of genebank-sourced genetic resource collections of lettuce for resistance to *N. ribisnigri* is both time consuming and expensive. A strategy commonly used to rationalize the problem is through the generation of core collections (Brown 1989, 1995; Reeves et al. 2012; van Hintum et al. 2000). These aim to represent the available variation in the species gene pool in a smaller set of contrasting accessions, minimizing the cost of genetic conservation. Examples of core collections include pea (*Pisum sativum* L.) (Ambrose and Coyne 2009), maize (Abadie et al. 1999; Li et al. 2004), and *Brassica oleracea* (Walley et al. 2012), and examples of lettuce core collections have been described (Cid et al. 2012, McCreight 2008; Simko and Hu 2008; van Treuren and van Hintum 2009). Lettuce is an inbreeding crop, with genebank accessions being predominantly homozygous, which reduces within accession phenotypic variation and makes genotyping less complicated.

The genus *Lactuca* is a member of the Asteraceae or Compositae family, characterized by their composite flowers. The total gene pool can be subdivided based on inter-fertility. The primary gene pool of lettuce is made up of the cultivated form (*L. sativa*) and different morphotypes of the ‘wild species’ *L. serriola* that are inter-crossable producing fertile F_1_ progeny. The secondary gene pool includes the wild species *L* *actuca saligna* and *L. virosa* (Koopman et al. 1998). These can be crossed with difficulty to *L. sativa* using the wild species as the female parent; however, bridging crosses as described above or embryo rescue is often employed (Maisonneuve et al. 1995; Maisonneuve 2003). These techniques are also used (with great difficulty) to access genetic variation in the tertiary gene pool (Lebeda et al. 2007).

In this paper, we describe the publicly available UK Vegetable Genetic Improvement Network lettuce diversity set, with an associated panel of next-generation sequencing-derived single nucleotide polymorphism (SNP) markers. The SNP panel was converted to breeder-friendly ‘Kompetitive Allele Specific PCR’ (KASP™) markers and used to assess population structure and phylogenetic relationships in the diversity set. In addition, we screened the diversity set for resistance to the currant-lettuce aphid *N. ribisnigri* to identify potential sources of new resistance factors effective against the aphid. Phenotype and genotype data were used to perform genome-wide association analyses to identify SNPs (expressed sequence tags (ESTs)) significantly linked to the observed resistance. The diversity set and its genomic tools are then placed in the context of how to take these data forward.

## Materials and methods

### Lettuce diversity set (DS)

The 96 accessions of the lettuce DS are an extension of 19 accessions selected from the lettuce collection at UK Vegetable Genebank, Wellesbourne, UK, used previously to quantify nitrate content (Burns et al. 2011) and post-harvest discolouration (Atkinson et al. 2012). A further 77 accessions were selected from the international *Lactuca* collection at Centre for Genetic Resources Netherlands (CGN) using the core selector tool (http://www.wageningenur.nl/en/Expertise-Services/Legal-research-tasks/Centre-for-Genetic-Resources-the-Netherlands-1/Expertise-areas/Plant-Genetic-Resources/Research-at-CGN/Core-collections/Core-selections.htm). Restrictions selected 17 wild species that were sexually compatible with *L. sativa*. This resulted in a DS of 96 accessions, representing diversity in crop type, geographical origin, and phenotype. The DS contains four parents of two mapping populations. The 96 accessions are described in detail in Supplementary Table S1. Accessions are available under a standard MTA and a cost recovery charge.

## Phenotyping resistance to *N. ribisnigri*

### Plant growth conditions

For each of the 96 DS accessions, four seeds were sown in a single pot in F2+s compost (Levington, UK) and kept at 18 °C 16L:8D in a randomized order in insect-proof growth chambers. After 2 weeks, seedlings were transplanted into 9-cm pots for aphid screening. This process was repeated on five occasions; sowing dates are as follows: 1 February 2011, 9 March 2011, 4 April 2011, 4 May 2011, and 16 June 2011. The experiment was arranged in an alpha design with ten replicates, each containing 8 blocks of 12 pots. This design ensured pairs of lines occurred together in the same block at most twice, allowing adjustments to be made in the analysis for spatial variation in levels of infestation. However, on the first occasion, variable germination was experienced resulting in two or more seedlings only being available for 28 lines, with no seedlings for 48 lines. A revised alpha design was generated using 12 blocks of either 6 or 7 pots each, the 12 blocks being grouped into 2 replicates of 6 blocks with 20 single replicate lines allocated across blocks. Eight replicates of the original alpha design (replicates containing 8 blocks of 12 pots each) were used for the four subsequent occasions, two replicates being used on each occasion, with spaces left in blocks where the allocated line failed to germinate. Lines were therefore replicated between 4 and 10 times within the experiment.

### *N. ribisnigri* inoculation

Five-week-old plants were inoculated with five newborn nymphs (1–2-day-old) of *N. ribisnigri* Nr:0 clone 4850a (derived from a founding mother collected on September 2003 from a lettuce field, Lincolnshire, UK), cultured previously on *L. sativa* cv. Pinokio (lacking *Nr*). Clone 4850a does not reproduce on cultivars possessing *Nr*. Inoculated plants were covered individually with micro-perforated polypropylene bags (200 mm × 500 mm) and arranged in an alpha design on a single shelf in a controlled environment room maintained at 20 °C, 16L:8D. After 3 weeks, aphid numbers on each plant were recorded (alates and apterous (apterous included nymphs)) over a 2-day period.

## Statistical analyses

For each accession, variance components were estimated and predicted means calculated for aphid counts (alates, apterous (including nymphs), and total aphids), using restricted maximum likelihood (REML) (Patterson and Thompson 1971). For all count data, a square root transformation (with an added constant of 0.375) was applied prior to analysis to allow the homogeneity of variance assumption to be satisfied. Data interpretations were made using the predicted means and 5 % least significant difference (LSD) values. Variation in the replication levels, and the complex blocking structure used, meant that there was considerable variation in the LSD values for different pairwise comparisons between accessions. The significance of reported differences is conservative as the maximum LSD at 5 % was used. All statistical analyses were performed using GenStat (VSNI, UK).

## Genetic analyses

### DNA extraction

Total DNA was isolated from young true leaves using DNeasy plant Maxi Kits (Qiagen Inc., UK) following manufacturer’s guidelines and diluted to 100 ng μl^−1^ using TE (pH 8.0) and stored at −20 °C.

### RNA preparation

Tissues from leaf, root, and stalk were harvested separately and flash frozen in liquid nitrogen for each of three individual plants of *L. sativa* cultivars Iceberg and Saladin. These accessions are parental lines of a recombinant-inbred line population (Pink 2004, 2009). Total RNA was isolated from each tissue sample using Plant RNeasy Mini Kits (Qiagen Inc., UK) following manufacturer’s instructions and quality assessed using BioAnalyzer (Agilent, UK).

### Illumina transcriptome sequencing

For cultivars Iceberg and Saladin, total RNA from individual tissue samples was pooled and Oligo(dT) selection performed twice using Dynal magnetic beads (Invitrogen, UK). Illumina libraries were prepared using TruSeq Stranded mRNA kit v5 (Illumina Inc., San Diego) following manufacturer’s protocol (15018818 A). Cultivar-specific libraries were multiplexed using six-nucleotide barcoded adapters and randomly assigned to two lanes. Seventy base paired-end sequence reads were generated for these libraries using an Illumina Genome Analyzer IIx and score read quality assessed using CASAVA v1.8 (Illumina Inc., San Diego).

## Molecular markers

### Identification of SNPs between Saladin, Iceberg, and ESTs

A reference sequence database was constructed comprising 76,043 ESTs from the CLS_S3_Sat.assembly (L. sativa|CAP3:100/95) database (http://cgpdb.ucdavis.edu/cgpdb2/est_info_assembly.php) supplementary Table S2. Illumina transcriptome reads were aligned to reference EST sequences using Bowtie v0.11.3 (Langmead et al. 2009), and consensus sequences corresponding to each reference sequence were generated for each accession using SAMtools v1.4 (Li et al. 2009). A custom Perl pipeline identified and filtered putative SNP loci between consensus accession sequences. Loci were discounted if base calls had very low coverage or low sequencing quality in either accession (Phred score < 33). To increase the likelihood of SNPs being from unique genomic regions, and therefore likely to be amenable to unambiguous PCR assay, 150-nt fragments centred on each putative SNP were extracted from consensus sequences and aligned to the reference database using BLAST (Altschul et al. 1990). Fragments were discounted unless aligning uniquely to a single sequence within the reference set with >98 % identity. After filtering, 1393 putative SNP loci were ranked for conversion to KASP™ assays in decreasing order of the lower Phred score of the SNP locus base calls from the two accessions. From this group, we selected a panel of 682 fragments to design KASP™ assays. The 150-nt fragments were labeled non-control representing unique sequences (*n* = 678) and internal control for matching pairs of sequence that aligned to different EST contigs and contain alternate SNP bases (*n* = 4).

### Lettuce-specific KASP™ assay markers (LKAMs)

The selected 682 SNP sequences were converted to KASP™ marker assays using the LGC Genomics (Hoddesdon, UK) ‘KASP™-On-Demand’ assay design service (Table S3) and are referred to here as LKAMs. LKAM genotyping was performed by LGC Genomics using 10 ng of supplied total DNA template, with four independent DNA samples each of cultivars Saladin and Iceberg. Quality and distribution of SNP calls across individuals were assessed using SNPViewer v1.99 (LGC Genomics). BLASTn (Altschul et al. 1990) was used to assign LKAM sequences to loci in the *L. sativa* pseudo-chromosome assembly ‘Lsat_1_v4’ (accessed 24 September 2012) from U.C. Davis Lettuce Genome Resource (https://lgr.genomecenter.ucdavis.edu/) with SNPs positioned in unassembled genomic regions being assigned to an arbitrary group 10.

### Hardy-Weinberg equilibrium (HWE), polymorphism information content (PIC), and genome-wide *r*^2^

PowerMarker v3.25 (Liu and Muse 2005) was used to assess LKAMs for departure from HWE using Fisher’s exact test (Fisher 1922) (10,000 permutations) to test for significance; calculate LKAM PIC values; and pairwise linkage disequilibrium (LD) estimated using the genetic correlation coefficient *r* ^2^ (Devlin and Risch 1995). A LD correlation matrix was assembled using marker assembly positions.

#### Phylogenetic analysis

Phylogenetic relationships between accessions were explored using LKAM genotype data. Data were first filtered to remove loci with heterozygotic, uncertain, un-scored assay calls in >20 % of accessions. Heterozygotic and uncertain calls in remaining loci were treated as missing data in subsequent analyses. The filtering removed cv. Lilian (GRU005491) and *L. saligna* (CGN05308). Phylogenetic analyses were performed using maximum likelihood (1000 bootstrap iterations each) implemented in MEGA v5.2.2 (Tamura et al. 2011). The majority of commercially available cultivars share common ancestry at some point in their pedigrees, with evidence of frequent intercrossing among breeding lines (Mikel 2007, 2013). Therefore, to complement phylogenetic analyses, we generated a split decomposition using the Neighbour-Net algorithm (Bryant and Moulton 2004) implemented in SplitsTree4 (Huson 1998) to explore non-tree-like relationships among individuals and infer putative historical intercrossing within clades.

### Genetic structure of the lettuce diversity set

LKAM data for 94 accessions used in phylogenetic analyses were used for Bayesian population structure analyses using STRUCTURE v2.3.3 (Pritchard et al. 2000a). To identify the number of sub-populations present (*K*), a burn-in period of 100,000 Markov Chain Monte Carlo iterations and 300,000 run-length were implemented using an admixture model following Hardy-Weinberg equilibrium and correlated allele frequencies. For each simulated value of *K* (for *K* = 1–10), four runs were repeated independently. To explore population structure in the *L. sativa* accessions, the STRUCTURE analyses were repeated with wild *Lactuca* species omitted. The same parameter settings were implemented for each simulated value of *K* (for *K* = 1–6) with four runs repeated independently. The python script structureHarvester.py v0.6.92 (Earl and vonHoldt 2012) was used to summarize STRUCTURE output. This script generates Δ*K* values using the method described by Evanno et al. (2005) to estimate the correct underlying *K*: briefly, *L*(*K*) = average of LnP(*D*) from STRUCTURE runs per *K*, *L*′*K* = *L*(*K*)_*n*_ − *L*(*K*)_*n*−1_, *L*″(*K*) = *L*′(*K*)_*n*_ − *L*′(*K*)_*n*−1_, Δ*K* = [*L*″(*K*)]/Stdev. Principal component analyses were performed using the R package Genome Association and Prediction Tool (GAPIT) (Lipka et al. 2012) and ‘prcomp’ function in the base R stats package (R Core Team 2014).

### Identification of putative SNPs linked to *N. ribisnigri* resistance

Two association methods were used to identify LKAMs linked to variation in *N. ribisnigri* count data observed in the lettuce DS:

## Kruskal-Wallis (rank-sum) test

The non-parametric Kruskal-Wallis test (Kruskal and Wallis 1952; Lehmann 1975) was used to assess the effect of genotype at each SNP on mean phenotype value using JoinMapv4 (Van Ooijen et al. 1993). The Kruskal-Wallis test statistic (*K*) was generated for each test, acting as a guide to SNP effect. Values for *K* are distributed approximately as a chi-square distribution with 1 *df* for the two-genotype classes. A stringent *P* value of ≤0.005 was used to minimize type I error (van Ooijen 2009).

## Linear mixed model

LKAM genotype data and *N. ribisnigri* phenotype data (no. alate, no. apterous, and total aphid count) were used for genome-wide association studies (GWAS) using GAPIT (Lipka et al. 2012) (http://www.maizegenetics.net/) in R (R Development Core Team 2014). GAPIT implements Efficient Mixed Model Analysis (EMMA) (Kang et al. 2008) and was used to fit the following mixed effects model:

$$ Y=X\beta +Zu+e $$

where *Y* is a vector of phenotype, *X* is a matrix of covariates (including SNP to test), and *ß* is a vector of fixed effects fitting *X* to *Y* and includes SNP, population structure (from *Q* matrix), and the intercept. The vector *u* contains the random effect of individual on phenotype and *Z* is a design matrix (analogous to *X*) but comprising 1’s and 0’s linking *u* to *Z*, with *e* as the residual error (Lipka et al. 2012). To account for possible spurious associations due to population structure, and cryptic relationships between individuals, genomic control was implemented using a *Q* matrix for *K* = 3 from STRUCTURE (Pritchard et al. 2000a) and a Kinship covariance matrix (van Randen 2008), respectively (Yu et al. 2006). Marker/trait associations were deemed significant if *P* ≤ 0.01. A final filter was applied to select SNPs that gave significant associations for both methods.

## Results

### Production of a lettuce diversity set

**Fig. 1:**
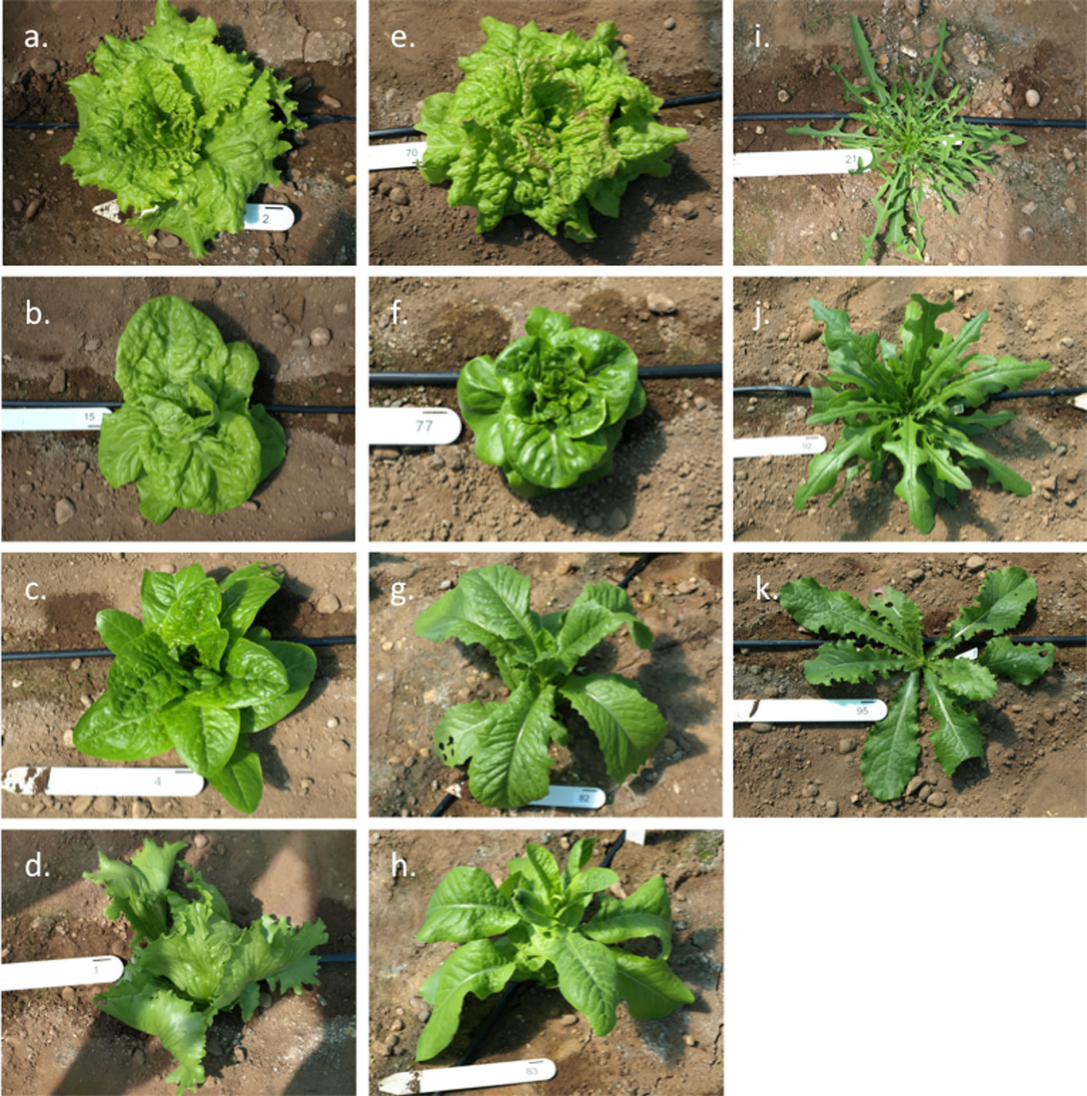
Morphological variation in the lettuce DS. **a**–**h** *L. sativa* crop types. **a** Batvian. **b** Butterhead. **c** Romaine/Cos. **d** Crisp/Iceberg. **e** Cutting/leaf. **f** Latin. **g** Oilseed. **h** Stem/stalk. **i**–**k** Wild *Lactuca* species. **i** *L. saligna*. **j** *L. serriola*. **k** *L. virosa*

The lettuce DS comprises 96 accessions sourced from Warwick Genetic Resources Unit and the Centre for Genetic Resources Netherlands. The collection represents variation in *L. sativa* crop types and the primary and secondary gene pool wild *Lactuca* spp. (Fig. 1 and Supplementary Table S1; http://www2.warwick.ac.uk/fac/sci/lifesci/research/vegin/lettuce/diversityset/). Accessions are inbred and produce good seed yields, suitable for use in replicated trials for phenotype analyses.

The lettuce DS does not overlap with other *Lactuca* collections such as those previously assembled (Cid et al. 2012; McCreight 2008; Simko and Hu 2008; van Treuren and van Hintum 2009) but serves to complement these resources for *Lactuca* research and breeding.

### Lettuce-specific KASP™ assays

Illumina RNA-seq reads from cultivars Saladin and Iceberg were assembled against 76,043 EST sequences (CLS_S3_Sat.assembly [http://cgpdb.ucdavis.edu/cgpdb2/est_info_assembly.php]), and 1393 EST sequences containing SNPs were selected, from which 682 were converted to LKAMs (Supplementary Table S3). Using BLASTn (Altschul et al. 1990), LKAM sequences were assigned loci on the Lsat-1_v4 *L. sativa* pseudo-chromosome assembly (http://lgr.genomecenter.ucdavis.edu) with 586 assigned to pseudo-chromosomes 1–9, and 96 located on genomic contigs not assembled in pseudo-chromosomes being assigned to an arbitrary group 10 (Supplementary Table S4 and Fig. S1). The SNPs provided an average inter-marker distance of 5.219 mbp (5219.7376 kbp) with a minimum distance of 54 bp and a maximum distance of 7.55 mbp (7558.3322 kbp).

### Genetic diversity in the lettuce diversity collection

**Fig. 2:**
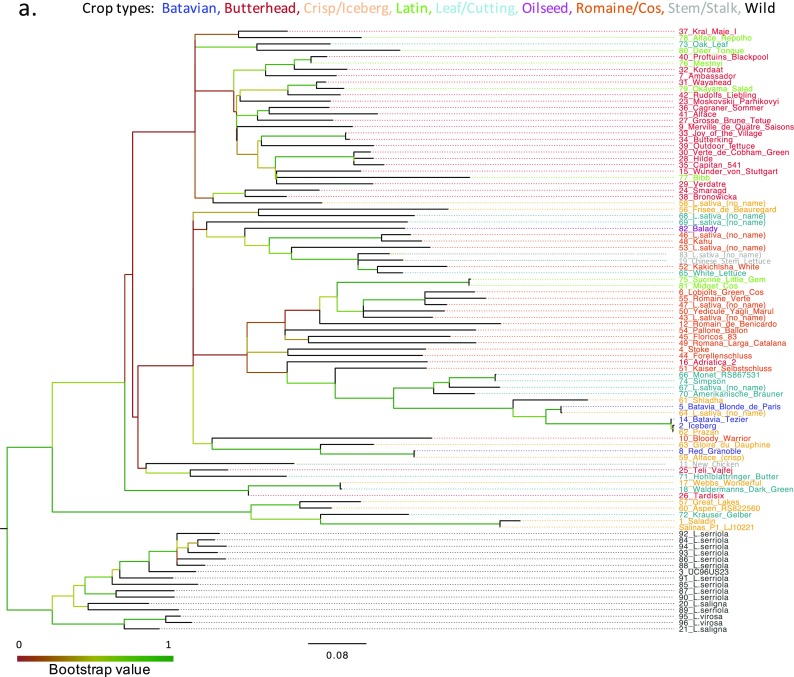

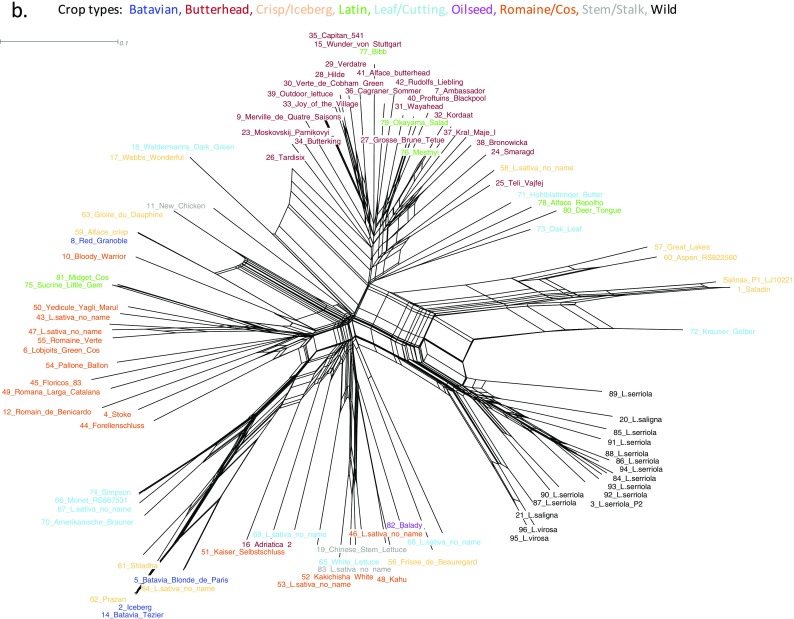
Genetic relationships in the lettuce DS. **a** Dendrogram of relationships between 96 lettuce accessions generated by maximum likelihood using 653 LKAMs. Branches were coloured according to bootstrap support, and line names are coloured according to morphotype. Cluster I: wild *Lactuca* species. Cluster II: domesticated species, sub-divided into cluster IIa (Butterhead types), cluster IIb (Cos/Romaine types) and cluster IIc (an evolutionarily distinct clade containing the Saladin type). **b** Neighbour-Net split graph showing inferred evolutionary relationships and likely recombination events among accessions

The 682 LKAMs were used to genotype the lettuce DS; of these, 29 were found to be monomorphic. Of the remaining 653 LKAMs, 244 (37.36 %) were monomorphic in the wild species, yet polymorphic in the crop types, and 6 LKAMs (0.92 %) were polymorphic in the wild species and monomorphic in the crop types, 2 LKAMs having one base monomorphic in the crop compared to the alternate base being monomorphic in the wild species. Overall levels of heterozygous calls in the collection were low, ranging between 0.59 and 25.37 % in the Russian cv. Mestyni (possibly derived from a landrace), $\overset{-}{x}$ = 1.91 % (Supplementary Fig. S2a). This is consistent with the inbreeding nature of this crop. The LKAMs were assessed for departure from Hardy-Weinberg equilibrium; using an exact test, 10 LKAMs were significant at *P* = <0.01, 3 at *P* = 0.001. These also had low PIC values. PIC values ranged between 0.021 and 0.375, with 61.92 % between 0.300 and 0.374, and 9.75 % were 0.375, $\overset{-}{x}$= 0.296 (Supplementary Fig. S2b, c). LD patterns across the genome were estimated using the disequilibrium coefficient (*r* ^2^) for multi-allelic pairwise comparisons (Hill and Robertson 1968). When *r* ^2^ values were plotted relative to inter-marker distances between pairs of loci, LD decays rapidly with distance (Supplementary Fig. S3); this is apparent when all pairwise comparisons are plotted as a heat map (Supplementary Fig. S4), since LD blocks are small and dispersed across the genome. Genotype data for 653 polymorphic LKAMs was used to assess genetic diversity captured by the collection, giving a better understanding of their genetic relationships. Genetic dissimilarities based on maximum likelihood estimates were used to construct a dendrogram (Fig. 2a).

The dendrogram separated the wild species (cluster I) from domesticated cultivars (cluster II) with strong support, and the cultivars were roughly clustered into morphological groupings in a weakly supported ‘comb’ with some strongly supported sub-clades. Iceberg and Saladin have the highest apparent genetic dissimilarity among cultivars, as a consequence of all LKAMs being polymorphic between them. Genotype data were then used to construct a split decomposition Neighbour-Net phylogenetic network (Fig. 2b) showing inferred evolutionary relationships and likely recombination events among accessions.

### Genetic structure in the lettuce diversity set

**Fig. 3:**
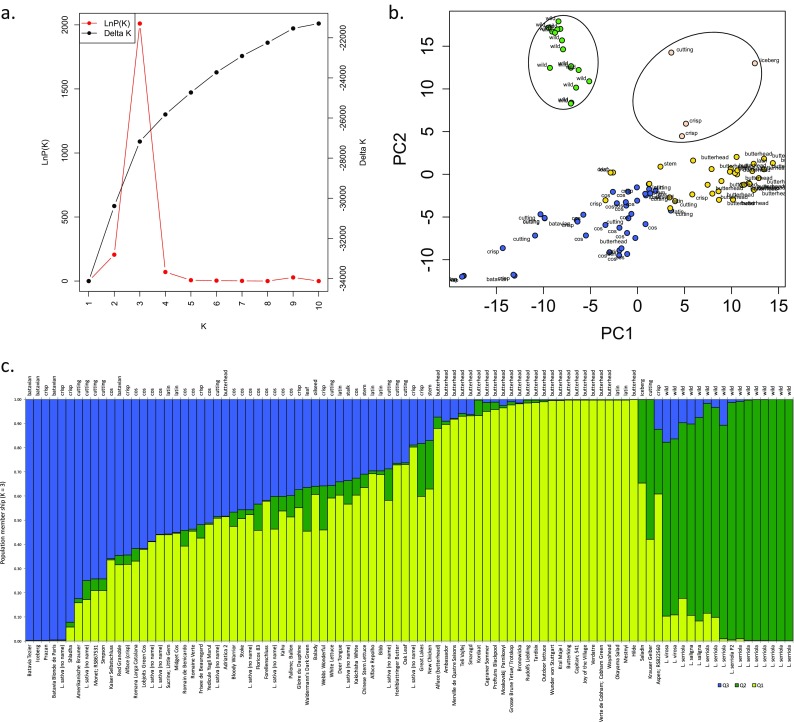
Population structure in the lettuce DS. **a** Δ*K* and mean LnP(*K*) derived from STRUCTURE output; *K* = 3 was chosen as the optimal *K* value. **b** Plot of first two eigenvectors from principal component analysis. Three sub-populations are coloured: *green* wild species, salmon: distinct sub-clade. Remaining domesticates are divided into *yellow*: Butterhead-like, *blue*: remaining accessions, predominantly Cos/Romaine types. **c** Bar plot of sub-population membership when *K* = 3 (Q1, Q2, Q3). *Vertical bar* segments are proportional to values of *Q* which together equal 1.0 (Σ_k_ *q*_k_ = 1). *Bars* labeled by lettuce name (primary *x*-axis, *bottom*) and lettuce crop type (secondary *x*-axis, *top*)

Underlying population sub-structure was investigated using STRUCTURE v2.3.3 (Pritchard et al. 2000a), in conjunction with the python script structureHarvester.py v0.6.92 (Earl and vonHoldt 2012) to identify a main Δ*K* peak at *K* = 3, suggesting three main sub-populations in the diversity set (Fig. 3a, c). Population sub-structure reflected that of the dendrogram in Fig. 2, these groupings were also resolved by the first two eigenvectors from principal component analysis (Fig. 3b).

The three inferred sub-populations are broadly the wild species (*L. serriola*, *L. saligna*, and *L. virosa*), a clade containing three Batavian and three crisp-head types, and a group of accessions encompassing Butterhead and Latin types. The remaining cultivars appear to be admixtures between these populations to varying degrees.

The cultivars that appear to share the greatest proportion of ancestry with the wild species clade are Salinas and Saladin (synonyms used in the USA and Europe, respectively (Grube et al. 2005)), Great Lakes. These accessions have pedigrees that contain introgressions from wild species. For example, Salinas, and its selection Saladin, were selected from the self-pollinated progeny of the cross Calmar × 8830(F_9_). Calmar is Great Lakes type with tip-burn resistance and downy mildew resistance acquired from a distant cross in its pedigree involving *L. serriola* (Welch et al. 1965, Ryder 1979, Mikel 2007). Line 8830 was an advanced breeding line of the Vanguard type, derived from a cross between breeding line 4157 and line 5192 (in its pedigree is a cross between (*L. serriola* × *L. serriola*) × *L. sativa* hybrid with an *L. virosa* accession (Thompson and Ryder 1961, Mikel 2007). Interestingly, line 4157 also has line 14,787 in its pedigree, from which the variety Great Lakes (has *L. serriola* derived downy mildew resistance) was selected (Thompson and Ryder 1961). The accessions Aspen: RS822560 and Krauser Gelber may also share pedigrees that involved wild species; these are still to be determined. The remaining cultivars appear to have been extensively intercrossed, which may be expected during the development of commercial breeding lines, although a clear division between Romaine/Cos and Butterhead types is still very apparent, possibly due to separate breeding programs for these lettuce types.

### Quantifying resistance to *N. ribisnigri* in the lettuce DS

**Fig. 4:**
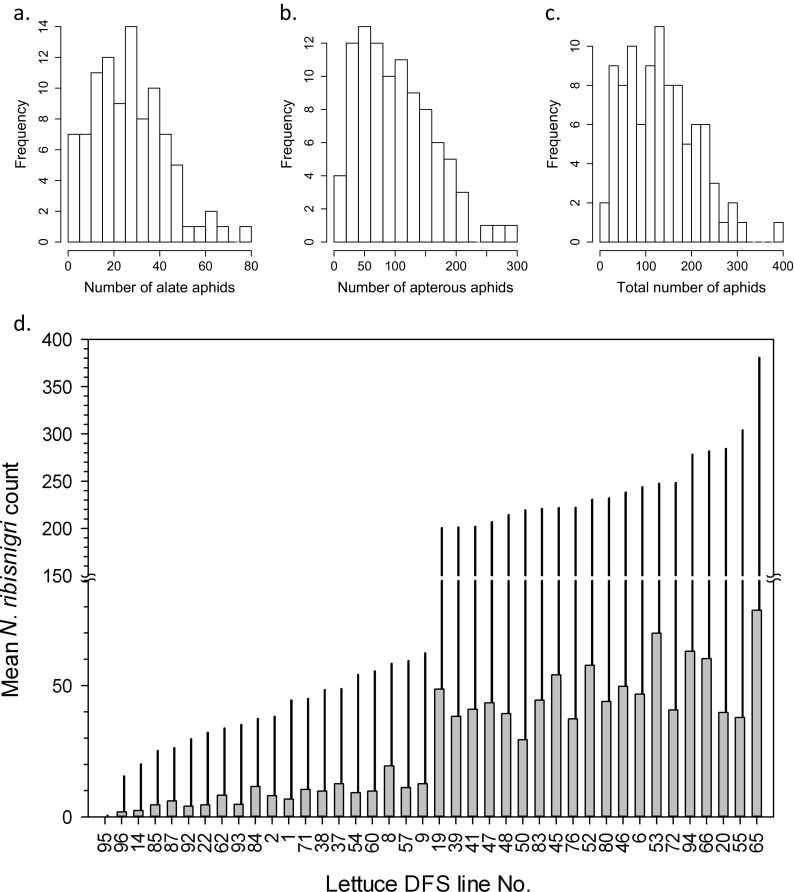
Frequency distributions of mean *N. ribisnigri* count data for the lettuce DS. **a** Alates. **b** Apterous. **c** Total number of aphids (alate + apterous). Data are back-transformed REML means. **d** Mean numbers of alate (*grey bar*) and total number of *N. ribisnigri* (*black needle*). For clarity, the 40 lines illustrated are taken from the extremes of the total distribution (insert). All data were recorded 3 weeks post-inoculation

To determine the degree of resistance to *N. ribisnigri*, the 96 lines were inoculated with *N. ribisnigri* clone 4850a (cannot survive on lettuce possessing *Nr*). At 3 weeks’ post-inoculation, plants were inspected and numbers of alate (winged) and apterous (non-winged) *N. ribisnigri* were counted; the total number of aphids on each accession was the sum of the two groupings. Figure 4 summarizes frequency distributions of aphid count data across the lettuce DS.

There was little effect of replicate, block and plot on numbers of *N. ribisnigri* per line. However, a significant effect of plant line was seen on the total numbers of aphids (Wald_[95]_ = 581.46, *P* < 0.001, n.d.f. 95 and d.d.f. 641.4), numbers of alates (Wald_[95]_ = 442.49, *P* < 0.001, n.d.f. 95 and d.d.f. 643.3) and numbers of apterae (Wald_[95]_ = 539.85, *P* < 0.001, n.d.f. 95 and d.d.f. 639.5; see Table S5 for REML variance components). The estimated population mean for the number of alate aphids was 28.676 with a range of 0.18–78.66. The estimated population mean for total number of aphids was 132.327, with a range of 0.6–380.7 aphids.

To compare resistance of lettuce lines to *N. ribisnigri*, we focused on the mean total number of aphids present and mean number of alate aphids in that group. Alates were chosen as they are the migrating morph (winged) and are considered the most sensitive to host plant differences, due partly to increased numbers of receptors on their antennae compared to apterous individuals (Bromley et al. 1979). From REML analyses, accession 95 (*L. virosa*, CGN15677) was most resistant, having the lowest mean total number of *N. ribisnigri* ($\overset{-}{x}$ = 0.6 aphids); of these, 0.18 were alates (back-transformed data). This line was significantly different (*P* = 0.001) to all other accessions, Fig. 4d. By contrast, accession 65 (*L. sativa*, white cutting lettuce) was most susceptible, with 78.66 alates and 380.7 aphids in total (back-transformed data), supporting 634 times more aphids compared to line 95.

The proportions of phenotypic variance explained by genetic variance (heritability) were reasonably high for each trait: alate (0.562), apterous (0.636), and total aphid count (0.674), suggesting aphid numbers are indeed influenced by host plant genotype (Supplementary Table S5).

### KASP™ markers linked to *N. ribisnigri* resistance

To explore the genetic basis of variation in *N. ribisnigri* resistance, associations between LKAM genotypes and aphid count data were quantified using two approaches, a non-parametric (Kruskal-Wallis) test and a linear mixed model test. The Kruskal-Wallis test was first used to estimate the effect of genotype at each SNP on mean phenotype value for numbers of alate, apterous and total *N. ribisnigri*. A value for the test statistic (*H*) was generated for each test with an associated *χ* ^2^ *P* value, acting as a guide to the SNP effect. A stringent *P* value was used as a cutoff for putative associations, since no account for population sub-structure was implemented for this method (Table S6). Numbers of significant SNPs (*P* ≤ 0.005) associated with counts for alate, apterous and total aphids were different, with 15 SNPs for alate, 10 for apterous and 13 for total aphid number, with 9 SNPs shared between the three-phenotype groupings. The alate group had three unique SNPs and shared three SNPs with the total aphid group. By contrast, the apterous group had one SNP shared with total aphid count but this was absent from the alate group. Interestingly, for each phenotype, it was the Iceberg-like SNP that was associated with a decrease in mean count, apart from markers LS1_242 and LS1_98, with the Saladin-like SNP associated with a decrease in mean counts.

Linear mixed model association analyses were performed using EMMA described by Kang et al. (2008), implemented in the R package GAPIT (Lipka et al. 2012). The fixed effect of SNP genotype was fitted after random effects of population sub-structure and kinship on phenotype were accounted for, providing a form of genomic control. Inspection of quantile–quantile plots suggested that implementing genomic control removed many spurious associations (Supplementary Fig. S5). The SNPs significantly associated with resistance were found to be distributed across a number of pseudo-chromosomes, with no clear spike as is commonly seen in other GWAS studies when a trait is linked to a SNP or gene of major effect (Atwell et al. 2010; Cockram et al. 2010; Huang et al. 2010). This may reflect the quantitative nature of the resistance observed. When *P* values for each SNP were plotted in relation to the genome assembly, clear patterns were observed, with seven SNPs being significant for all three-phenotype classes (Supplementary Fig. S5 and Table S7) on pseudo-chromosomes 1, 4, 5 and 9, with others that were significant for a single or two phenotypes.

SNPs significantly associated with each aphid morph type were compared to the significant SNPs identified in the Kruskal-Wallis tests. Six SNPs were significant in both tests (Table 1); the other SNPs that were significant in either test alone showed clear morph-specific associations (Supplementary Table S8). Interestingly, the alate phenotype had a greater number of unique SNPs that were not significant in the apterous, total aphid or combined groupings.

**Table 1:** LKAMs significantly associated with *N. ribisnigri* phenotypes in both Kruskal-Wallis and mixed model tests

Phenotype	LKAM (SNP)	Alleles	Pseudo-Chr.	Position (bp)	Kruskal-Wallis test			Mixed model GWAS		
					Alate *P* value	Apterous *P* value	TAC *P* value	Alate *P* value	Apterous *P* value	TAC *P* value
Apt.	LS1_400	G/***A***	1	207,327,226	0.1*	0.005	0.01*	0.05314**	0.0082	0.01269**
Alate	LS1_98	***T***/C	3	49,575,327	0.005	0.01*	0.005	0.0046	0.01158**	0.01127**
Alate, Apt. and TAC	LS1_51	A/***C***	4	72,830,000	0.0001	0.0005	0.0001	0.0003	0.0002	0.0001
Alate, Apt. and TAC	LS1_729	T/***C***	5	127,725,380	0.0005	0.001	0.0005	0.0080	0.0034	0.0025
Alate	LS1_381	C/***T***	8	206,453,742	0.0001	0.001	0.0005	0.0053	0.05687**	0.04291**
Alate	LS1_695	G/***A***	8	206,512,113	0.0001	0.005	0.001	0.0096	0.07001**	0.05856**

The allelic variant associated with a decrease in aphid number is presented in bold-italics

*Apt.* apterous, *TAC* total aphid count

*P < 0.005 Kruskal-Wallis test, not significant

**P < 0.01 linear mixed model GWAS, not significant

The LKAMs are linked to annotated lettuce ESTs in the CLS_S3_Sat.assembly. To add to these putative annotations, sequences of the LKAMs identified in the association mapping were used in a BLASTn (Altschul et al. 1990) search of TAIR10 (Lamesch et al. 2012) to identify the closest matching *Arabidopsis* gene models. The LKAM LS1_51 on pseudo-chromosome 4 was the most significant SNP for alate, apterous and total aphid count phenotypes, using both association methods; alignment to TAIR10 returned At3g61540, a peptidase family protein similar to prolyl aminopeptidase (Table S8). The LKAM LS1_729 was also associated with the three aphid phenotypes. The marker sequence aligned to the *Arabidopsis* gene model At3g46290, encoding a HERCULES Receptor Kinase 1 (HERK1), belonging to the *Catharanthus roseus* RLK1 (CrRLK1)-like protein family (Supplementary Table S9).

LKAMs LS1_381 and LS1_695 were both significant for the number of alate aphids and are 58,371 bp apart, with strong linkage to each other (*r* ^2^ = 0.91) but not with SNPs on either side of this interval, suggesting that gene(s) affecting the number of alate morphs maybe linked to this interval. LS1_381 has sequence homology with At3g12750, a Zinc transporter (ZIP1), whereas LKAM LS1_695 aligns to At1g37130, encoding nitrate reductase 2 (NR2) (Supplementary Table S8). LS1_98 was also significantly associated with the number of alate morphs, and returned At2g25140, encoding a putative heat shock protein (HSP100) (Table S8). From the individual association tests (not significant for both tests), mixed model analyses identified LS1_256 and LS1_595 (*r* ^2^ = 0.71) as significantly associated with all aphid phenotype groups. These two markers delimit a 643,928-bp interval containing LS1_666 (*r* ^2^ = 0.72 and 0.86, respectively); however, LS1_666 was not significant for the KW or mixed model analyses, although the mixed-model associations were just above significance (*P* = 0.01), with alates *P* = 0.055, apterae *P* = 0.061 and total count *P* = 0.045.

Finally, there was a cluster of three LKAMS spanning 432,820 bp on pseudo-chromosome 9, two that were associated with the number of alates and the third with all groups.

The association analyses demonstrate the utility of the diversity set and the accompanying LKAM panel. One must remember that the associations identified are likely to be indirect associations (proxy SNPs) with genes in the vicinity of the markers; however, it is clear that resistance to *N. ribisnigri* has a polygenic nature and that differences in plant genotype seem to influence the proportion of morph types.

## Discussion

The lettuce DS was established in an attempt to maximize representation of genetic diversity in an amenable number of lines for routine study as a source of valuable alleles for lettuce breeding. The collection represents a structured sampling of genetic variation present in *L. sativa* domesticated accessions and the more diverse wild *Lactuca* spp. The wild ‘unadapted’ germplasm provides the opportunity to explore the presence of alleles not represented in cultivated germplasm that may be introgressed into domesticated species using conventional breeding strategies (Zohary 1991). Indeed, wild species have previously been used to improve disease/insect resistance and desirable morphological characteristics (Grube et al. 2005; Hand et al. 2003; Jeuken et al. 2008; Mikel 2007; Zhang et al. 2007).

Many marker types have been developed for lettuce, including AFLP, RFLP and SSRs. These have been used for linkage map construction and diversity assessment within *Lactuca* (Kesseli et al. 1994; Koopman et al. 2001; Syed et al. 2006; Truco et al. 2007; van Treuren and van Hintum 2009; van der Wiel et al. 1998) and QTL mapping (Grube et al. 2005; Jeuken et al. 2008; Zhang et al. 2007). However, developing linkage maps that provide even genome-wide marker distribution has been hampered by the large size of the lettuce genome (2.65 pg/1C ~2.5–2.7 Gb per haploid genome) (Arumuganathan and Earle 1991), lack of recombination and/or the number of polymorphic markers available, owing in part to difficulties establishing wide crosses and the inbred nature of the germplasm. Advances in next-generation sequencing and availability of reference sequence (http://lgr.genomecenter.ucdavis.edu) now provide a means to re-sequence mapping parents, aiding the discovery of large numbers of SNPs which can be physically positioned in genome sequence (Allen et al. 2011; Cortes et al. 2011; Rafalski 2002; Truco et al. 2013; Truong et al. 2012) and putative functions assigned to coding regions based on alignment with *Lactuca* EST databases (Simko 2009; Stoffel et al. 2012; Truco et al. 2007). These SNPs provide increased resolution for analysis of germplasm diversity (Hiremath et al. 2012), cryptic relationships between individuals and population sub-structure (Breseghello and Sorrells 2006; Pritchard and Rosenberg 1999; Simko and Hu 2008), providing a means of genomic control when undertaking association mapping in diverse often admixed populations (Devlin et al. 2001; Pritchard et al. 2000b; Simko and Hu 2008; Simko et al. 2009; Yu et al. 2006).

The SNPs identified in this work were selected by aligning transcriptome sequence from cultivars Saladin and Iceberg with 76,043 ESTs. SNP selection based on a small sample introduces ascertainment bias, since we will never be able to truly represent allele frequencies/distribution seen in the complete *Lactuca* gene pool. This bias will always be present when using crop type representatives, since these are sampled from a distorted sub-population that has been subjected to selection (Marth et al. 2004, Ganal et al. 2009). By contrast, one of the downsides of using diverse lines to call SNPs, e.g. wild species, is that they tend to have low frequencies in domesticated crop types, reducing their utility as markers if applied to other ‘cultivated’ lettuce mapping populations.

We converted the SNPs to KASP™ assays; these are attractive as a genotyping methodology. They are relatively cheap, reproducible and flexible in numbers to be assayed. The LKAM panel has good PIC values and represents a useable tool for diversity assessment across crop types. We demonstrated this by investigating phylogenetic relationships (Fig. 2a, b) and population sub-structure (Fig. 3a–c) in the diversity set. The SNPs separated wild species from domesticated accessions; the maximum likelihood dendrogram and Neighbour-Net split graph suggest there may have been an early domestication event followed by separation of accessions forming subtypes (morphotypes), with intercrossing between individuals within the two main subtype clades of Butterhead-like and Cos-like. These data suggest that many of the SNPs may have arisen before the domestication event (Morin et al. 2004); indeed, 409 of the 682 LKAM SNPs are polymorphic in the wild species accessions. It was interesting to note that 244 LKAMs segregated in the crop types and not the wild species. These may represent SNPs that have arisen post-domestication. However, a larger sampling of the wild species diversity would be needed to confirm this. Population structure analyses identified three main sub-populations (Fig. 1) in agreement with previously published work examining 54 cultivars and 6 wild species (Simko and Hu 2008). It is clear that assignment to morphological groupings based on crop type may differ to that suggested by the genetic relatedness and that morphological groupings alone should not be used as a covariate in linear mixed models to account for population structure. This is particularly true when marker-trait associations are to be estimated using traits that are correlated with crop type.

The utility of the lettuce DS was demonstrated by screening for resistance to currant-lettuce aphid *N. ribisnigri*, at different stages of development. The wild species *L. virosa* (CGN15677) was the most resistant to all aphid morph stages, consistent with *L. virosa* being the original source of *Nr* (Cid et al. 2012; Eenink et al. 1982a; McCreight and Liu 2012). CGN15677 may therefore possess the same *Nr* allele that has been incorporated into modern lettuce cultivars. However, since this line is more resistant than all other lines, some of which are expected to contain *Nr*, suggesting that this line may contain alternative or additional alleles that further enhance resistance.

The quantitative nature of the observed resistance was explored using both non-parametric and mixed model SNP/trait associations. In general, the Kruskal-Wallis test identified more significant associations compared to the mixed model method, even with a stringent significance level applied. This inflation in positive associations could be linked with the lack of genomic control to adjust for population sub-structure, as applied to the mixed model analyses. Uncorrected overestimates have been commented on previously when applying a form of rank-sum test to look for SNP associations (Atwell et al. 2010; Filiault and Maloof 2012). When the mixed models were run without genomic control, there were many false positives; on implementing genomic control using the *Q* and kinship matrices, the number of significant associations reduced (Fig. S3).

The SNPs significantly associated with aphid count data and the LD in the vicinity of those SNPs together define genomic intervals to look for candidate genes. Linkage disequilibrium in lettuce has been estimated as *r* ^2^ ~ 0.2 between 0.5 and 1 cM (Simko et al. 2009); this rapid decay of LD with distance was observed in this study, with LD limited to small segmental blocks. This may be due to high levels of historical recombination between breeding lines, as revealed by the spit decomposition analysis and the diverse nature of the germplasm, along with reduced marker coverage relative to the size of the genome. As a first step towards nominating candidates, putative functions of the significant SNPs were evaluated for potential to confer aphid resistance (Supplementary Table S8). The SNPs identified are thought to be indirectly associated with the causative SNP(s); therefore, increased marker density within these regions would be desirable. The association analyses identified SNPs linked to numbers of *N. ribisnigri* morph types and to total aphid count. Morph-specific resistance highlights the polygenic nature and complexity of plant genotype × aphid interaction, presenting the possibility of including morph-specific resistance as a tool for the control of this aphid. It would be interesting to assess if these SNP/trait associations would be observed for other aphid species (generalist versus specialist).

Patents protect the use of *Nr*, and its genomic location has not been described. The diversity set is anticipated to include lines that possess *Nr*. Since we found SNPs associated with resistance positioned across different chromosomes, we are unable to determine which SNPs may be linked to or represent *Nr*. In fact, it is possible that *Nr* has not been detected at all and a much higher density of SNPs may be required to do so. This is likely as it would have been desirable for breeders to select for recombination around *Nr* to remove non-crop-adapted *L. virosa* flanking sequence. It would be interesting to compare the observed resistance of lines to original cultivar release dates, i.e. pre- and post-introgression of the *Nr* allele, but we found this information to be not readily available. The associations on different groups also imply that, in addition to *Nr*, the diversity set contains lines with other resistance alleles that have not previously been described; these alleles therefore represent putative targets to pyramid for enhanced resistance.

The analyses presented may be used to complement future linkage mapping of *N. ribisnigri* count data using segregating populations that have been genotyped using the LKAMs, for example the Saladin × Iceberg RIL population (Pink 2004, 2009). This complementary approach was used to identify markers linked to lettuce dieback resistance and the association of *Tvr1* as the source of resistance (Simko et al. 2009).

Markers or candidate genes linked to aphid resistance will inform subsequent selection in pre-breeding programs; therefore, having a wider choice of ‘useable’ molecular markers distributed across the genome will help facilitate this (Collard and Mackill 2008; Lande and Thompson 1990; Staub et al. 1996). The new panel of EST-based LKAMs generated in this work is a public resource and should be valuable as a marker system to facilitate MAS in this crop, especially when the *L. sativa* reference genome has been completed.

In addition to lettuce (*Lactuca* spp.), *N. ribisnigri* infests a number of alternative herbaceous s including other commercial Asteraceae such as chicory (*Cichorium intybus* L.), endive (*Cichorium endive* L.) and some common weed species (Blackman and Eastop 1984). Markers linked to regions of resistance, or in the best case candidate genes, may form a basis for comparative studies for introducing resistance into other Asteraceae, to reduce the reproductive range of this pest.

## Conclusions

The VeGIN lettuce diversity set is a valuable breeding resource that captures wide genetic variation in *Lactuca* species and has a diverse range of morphological variation. This population is accompanied by an informative panel of lettuce-specific KASP™ markers that have been anchored in the *L. sativa* genome assembly and are amenable to cost-effective high-throughput genotyping or as smaller subsets for MAS. The genetic relationships in this population were determined allowing insights into the range of diversity captured and to serve as a means of accounting for population stratification during association analyses. The value of the diversity set for lettuce breeding research has been demonstrated by phenotyping for resistance to the economically important aphid *N. ribisnigri* biotype Nr:0, and several LKAM/ESTs associated with observed resistance have been nominated. This opens up the possibility for MAS of other alleles that may complement the allelic series at *Nr* or be used as a new source of resistance to be incorporated into a suitable integrated pest management programme to control this pest.

## Electronic supplementary material

Supplementary Table S1 VeGIN Lettuce Diversity set – list of lines and type.xlsx (EMS1) (PDF 59 kb)

Supplementary Table S2 Features of the CGP EST assembly CLS_S3_ESTs_Sat, EST library.pdf (EMS2) (PDF 40 kb)

Supplementary Table S3 Lettuce Specific KASP™ assay primer pairs.xlsx (ESM3) (XLSX 217 kb)

Supplementary Table S4 *Lactuca sativa* Lsat_1_v4 pseudo-chromosome assembly.xlsx (ESM4) (PDF 24 kb)

Supplementary Fig. S1 Position of LKAMS in assembly.pdf (EMS5) (PDF 202 kb)

Supplementary Fig. S2 Heterozygozity and PIC of LKAMS.pdf (EMS6) (PDF 274 kb)

Supplementary Fig. S3 LD decay with distance.pdf (EMS7) (PDF 166 kb)

Supplementary Fig. S4 Pairwise rsq in assembly.pdf (EMS8) (PDF 381 kb)

Supplementary Table S5 REML variance components.xlsx (EMS9) (PDF 30 kb)

Supplementary Table S6 Kruskal-Wallis association tests.xlsx (EMS10) (XLSX 49 kb)

Supplementary Fig. S5 Manhattan and Q-Q plots with genomic control.pdf (EMS11) (PDF 219 kb)

Supplementary Table S7 GAPIT GWAS.xlsx (EMS12) (XLSX 56 kb)

Supplementary Table S8 GAPIT GWAS other associations.xlsx (EMS13) (XLSX 55 kb)

Supplementary Table S9 Putative roles of the LKAM SNPs significant in both association tests.xlsx (EMS14) (PDF 67 kb)
