# Supplementary material for: Towards new sources of resistance to the currant-lettuce aphid (Nasonovia ribisnigri)
Source: Mol Breed. 2017 Jan 3;37(1):4. doi: 10.1007/s11032-016-0606-4 (PMC5209396; doi:10.1007/s11032-016-0606-4)
Supplement: Supplementary file 1 — VeGIN Lettuce Diversity set – list of lines and type.xlsx (EMS1) (PDF 59 kb) [file 11032_2016_606_MOESM1_ESM.pdf]

Table S1 VeGIN Lettuce Diversity Foundation set. Line number, Species and crop type are listed. Seed numbers prefixed by Warwick are internal codes, GRU are from The UK Vegetable Genebank, and CGN are from the Centre for Genetic Resources, the Netherlands.

Line no.	Seed No.	Species	Name	Type	Origin	Other traits scored	
1	Warwick LJ 09001	*L. sativa*	Saladin	Crisp/ Iceberg	–	Nitrate	Discoulouration
2	Warwick LJ 09002	*L. sativa*	Iceberg	Batavian	–	Nitrate	Discoulouration
3	Warwick LJ 08126	*L. serriola*	UC96US23	Wild serriola	–	Nitrate	Discoulouration
4	GRU002492	*L. sativa*	Stoke	Romaine/ Cos	GBR	–	Discoulouration
5	GRU001861	*L. sativa*	Batavia Blonde de Paris	Batavian	FRA	Nitrate	Discoulouration
6	GRU005492	*L. sativa*	Lobjoits Green Cos	Romaine/ Cos	GBR	Nitrate	Discoulouration
7	GRU005048	*L. sativa*	Ambassador	Butterhead	–	Nitrate	Discoulouration
8	GRU001228	*L. sativa*	Red Granoble	Batavian	GBR	Nitrate	Discoulouration
9	GRU004435	*L. sativa*	Merville de Quatre Saisons	Butterhead	FRA	Nitrate	Discoulouration
10	GRU001206	*L. sativa*	Bloody Warrior	Romaine/ Cos	GBR	Nitrate	Discoulouration
11	GRU001735	*L. sativa*	New Chicken	Stem/ Stalk	–	Nitrate	Discoulouration
12	GRU001683	*L. sativa*	Romain de Benicardo	Romaine/ Cos	GBR	Nitrate	Discoulouration
13	GRU005491	*L. sativa*	Lilian	Butterhead	GBR	Nitrate	Discoulouration
14	GRU004986	*L. sativa*	Batavia Tezier	Batavian	FRA	–	Discoulouration
15	GRU001783	*L. sativa*	Wunder von Stuttgart	Butterhead	–	–	Discoulouration
16	GRU013825	*L. sativa*	Adriatica 2	Butterhead	BGR	–	Discoulouration
17	GRU001881	*L. sativa*	Webbs Wonderful	Crisp/ Iceberg	GBR	Nitrate	Discoulouration
18	GRU001474	*L. sativa*	Waldermann's Dark Green	Leaf/ Cutting	CAN	Nitrate	Discoulouration
19	GRU004480	*L. sativa*	Chinese Stem Lettuce	Stem/ Stalk	CHN	Nitrate	Discoulouration
20	CGN20693	*L. saligna*	–	Wild	UZB	–	–
21	CGN05301	*L. saligna*	–	Wild	FRA	–	–
22	CGN05308	*L. saligna*	–	Wild	ISR	–	–
23	CGN09337	*L. sativa*	Moskovskij Parnikovyi	Butterhead	SUN	–	–
24	CGN04805	*L. sativa*	Smaragd	Butterhead	CZE	–	–
25	CGN04910	*L. sativa*	Teli Vajfej	Butterhead	HUN	–	–
26	CGN05136	*L. sativa*	Tardisix	Butterhead	NLD	–	–
27	CGN09331	*L. sativa*	Grosse Brune Tetue; Bruine Trotskop	Butterhead	CHE	–	–
28	CGN04613	*L. sativa*	Hilde	Butterhead	BEL	–	–
29	CGN04881	*L. sativa*	Verdatre	Butterhead	TUN	–	–
30	CGN04884	*L. sativa*	Verte de Cobham; Cobham Green	Butterhead	GBR	–	–
31	CGN04888	*L. sativa*	Wayahead	Butterhead	USA	–	–
32	CGN04807	*L. sativa*	Kordaat	Butterhead	NLD	–	–
33	CGN04756	*L. sativa*	Joy of the Village	Butterhead	ISR	–	–
34	CGN04540	*L. sativa*	Butterking	Butterhead	USA	–	–
35	CGN05140	*L. sativa*	Capitan [541]	Butterhead	FRA	–	–
36	CGN13295	*L. sativa*	Cagraner Sommer	Butterhead	AUT	–	–
37	CGN04916	*L. sativa*	Kral Maje I	Butterhead	CZE	–	–
38	CGN05193	*L. sativa*	Bronowicka [Inspektowa Bronowicka]	Butterhead	POL	–	–
39	CGN05959	*L. sativa*	Outdoor lettuce	Butterhead	SWE	–	–
40	CGN04521	*L. sativa*	Proftuins Blackpool	Butterhead	–	–	–
41	CGN19017	*L. sativa*	Alface	Butterhead	PRT	–	–
42	CGN05972	*L. sativa*	Rudolfs Liebling	Butterhead	DEU	–	–
43	CGN13383	*L. sativa*	–	Romaine/ Cos	BGR	–	–
44	CGN09375	*L. sativa*	Forellenschluss [Grazer Forellenschlus]	Romaine/ Cos	AUT	–	–
45	CGN16252	*L. sativa*	Floricos 83	Romaine/ Cos	USA	–	–
46	CGN05219	*L. sativa*	PI 212099	Romaine/ Cos	AFG	–	–
47	CGN05220	*L. sativa*	PI 206964	Romaine/ Cos	TUR	–	–
48	CGN05226	*L. sativa*	Kahu	Romaine/ Cos	IRN	–	–
49	CGN05240	*L. sativa*	Romana Larga Catalana	Romaine/ Cos	ESP	–	–
50	CGN04737	*L. sativa*	Yedicule Yagli Marul	Romaine/ Cos	TUR	–	–
51	CGN04904	*L. sativa*	Kaiser Selbstschluss	Romaine/ Cos	DEU	–	–
52	CGN04628	*L. sativa*	Kakichisha White	Romaine/ Cos	JPN	–	–
53	CGN04927	*L. sativa*	LAC 193/72	Romaine/ Cos	SWE	–	–
54	CGN04711	*L. sativa*	Pallone [Ballon]	Romaine/ Cos	ITA	–	–
55	CGN05236	*L. sativa*	Romaine Verte	Romaine/ Cos	TUN	–	–
56	CGN04581	*L. sativa*	Frisee de Beauregard [Reine des Glaces a Graine Noi	Crisp/ Iceberg	FRA	–	–
57	CGN05182	*L. sativa*	Great Lakes	Crisp/ Iceberg	USA	–	–
58	CGN13348	*L. sativa*	VIR 1532	Crisp/ Iceberg	UZB	–	–
59	CGN18629	*L. sativa*	Alface [IHR 10,011674]	Crisp/ Iceberg	PRT	–	–
60	CGN19088	*L. sativa*	Aspen [RS822560]	Crisp/ Iceberg	AUS	–	–
61	CGN13378	*L. sativa*	Shladha	Crisp/ Iceberg	DZA	–	–
62	CGN09359	*L. sativa*	Prazan	Crisp/ Iceberg	–	–	–
63	CGN09381	*L. sativa*	Gloire du Dauphine [Batavia Gloire du Dauphine]	Crisp/ Iceberg	FRA	–	–
64	CGN05227	*L. sativa*	PI 261617	Crisp/ Iceberg	ESP	–	–
65	CGN05837	*L. sativa*	White Lettuce	Leaf/ Cutting	TWN	–	–
66	CGN11446	*L. sativa*	Monet [RS867531]	Leaf/ Cutting	NLD	–	–
67	CGN04787	*L. sativa*	PI 269498	Leaf/ Cutting	PAK	–	–
68	CGN04797	*L. sativa*	PI 278091	Leaf/ Cutting	TUR	–	–
69	CGN18981	*L. sativa*	CNRIDG 201498 Cutting	Leaf/ Cutting	DZA	–	–
70	CGN05852	*L. sativa*	Amerikanische Brauner [Amerikaanse Roodrand]	Leaf/ Cutting	USA	–	–
71	CGN04618	*L. sativa*	Hohlblattringer Butter	Butterhead	DEU	–	–
72	CGN04642	*L. sativa*	Krauser Gelber [A Couper Fuille de Chene a Graine B	Leaf/ Cutting	DEU	–	–
73	CGN04707	*L. sativa*	Oak Leaf	Leaf/ Cutting	USA	–	–
74	CGN04849	*L. sativa*	Simpson	Leaf/ Cutting	FRA	–	–
75	CGN04858	*L. sativa*	Sucrine [Little Gem]	Latin	FRA	–	–
76	CGN05204	*L. sativa*	Mestnyi [VIR K1203]	Latin	RUS	–	–
77	CGN06018	*L. sativa*	Bibb	Latin	NLD	–	–
78	CGN18985	*L. sativa*	Alface Repolho	Latin	PRT	–	–
79	CGN18617	*L. sativa*	Okayama Salad	Latin	JPN	–	–
80	CGN04564	*L. sativa*	Deer Tongue	Latin	USA	–	–
81	CGN04696	*L. sativa*	Midget Cos	Latin	USA	–	–
82	CGN10975	*L. sativa*	Balady [IEE 217 Oilseed]	Oilseed	EGY	–	–
83	CGN04926	*L. sativa*	–	Stem/ Stalk	SUN	–	–
84	CGN05804	*L. serriola*	–	Wild	CHE	Nitrate	Discoulouration
85	CGN15670	*L. serriola*	–	Wild	ARM	–	–
86	CGN14278	*L. serriola*	–	Wild	HUN	–	–
87	CGN15737	*L. serriola*	–	Wild	GEO	–	–
88	CGN18668	*L. serriola*	–	Wild	ITA	–	–
89	CGN22054	*L. serriola*	–	Wild	KGZ	–	–
90	CGN15728	*L. serriola*	–	Wild	AZE	–	–
91	CGN15684	*L. serriola*	–	Wild	TUR	–	–
92	CGN05149	*L. serriola*	–	Wild	KEN	–	–
93	CGN09308	*L. serriola*	–	Wild	FRA	–	–
94	CGN05152	*L. serriola*	–	Wild	POL	–	–
95	CGN13355	*L. virosa*	–	Wild	ESP	–	–
96	CGN15677	*L. virosa*	–	Wild	RUS	–	–

The lettuce diversity set includes 19 accesions that were originally selected from the lettuce collection maintained by the UK Vegetable Genebank at Wellesbourne, UK. These were used in studies of genetic variation for nitrate content (Burns et al., 2011) and post harvest discolouration (Atkinson et al., 2012) in lettuce; the 24 original accessions were retained in the extended diversity set because the previous studies had shown they displayed significant phenotypic variation for agronomically/economically important traits. The additional 72 accessions were selected from the international Lactuca collection maintained by Centre for Genetic Resources Netherlands (CGN) using the core selector tool provided on the CGN website; for description of core selections see: (http://www.wageningenur.nl/en/Expertise-Services/Legal-research-tasks/Centre-for-Genetic-Resources-the-Netherlands-1/Expertise-areas/Plant-Genetic-Resources/Research-at-CGN/Core-collections/Core-selections.htm).

Country codes. GBR: United Kingdom; AFG: Afghanistan; ARM: Armenia; AUS: Australia; AUT: Austria; AZE: Azerbaijan; BEL: Belgium; BGR: Bulgaria; CAN: Canada; CHE: Switzerland; CHN: China; CZE: Czech Republic; DEU: Germany; DZA: Algeria; EGY: Egypt; ESP: Spain; FRA: France; GBR: United Kingdom; GEO: Georgia; HUN: Hungary; IRN: Iran (Islamic Republic of); ISR: Israel; ITA: Italy; JPN: Japan; KEN: Kenya; KGZ: Kyrgyzstan; NLD: Netherlands; PAK: Pakistan; POL: Poland; PRT: Portugal; RUS: Russian Federation; SUN: Union of Soviet Soc. Rep.; SWE: Sweden; TUN: Tunisia; TUR: Turkey; TWN: Taiwan, Province of China; USA: United States of America; UZB: Uzbekistan.

A note on further selection. PGW

The lettuce diversity set represents a starting point for trait evaluation. For many purposes, the full set with suitable replication is ideal; however, a reduced set may be required: a. limitted number of accessions can be used in one trial; b. accessions already identified as important, and need to include a further *n* individuals to complete a desired experimental design; c. want to maximise the diversity in a reduced subset e.g. GBS selection. We can produce a reduced set using one, or several criteria: phenotype distribution; morphology; passport data; geneotype data. From the genotype data we can refine individuals based on: the distribution of accesions and their *q* values in a *q*-matrix (STRUCTURE); we may want to select specific allele genotype combinations (QTL/ association). Many of these selection criteria can be performed using a spreadsheet. A number of software packages are available to assist, such as TASSEL (Bradbury et al., 2007) and Popmin (Hospital and Decoux, 2002). I have found that GGT2 (Van Berloo, 2008) is suitable for making reduced diversity sets, using the maximum diversity option. If there are certain individuals that are to be present, these can be pre-selected for inclusion.
