# Supplementary material for: Towards new sources of resistance to the currant-lettuce aphid (Nasonovia ribisnigri)
Source: Mol Breed. 2017 Jan 3;37(1):4. doi: 10.1007/s11032-016-0606-4 (PMC5209396; doi:10.1007/s11032-016-0606-4)
Supplement: Supplementary file 2 — Features of the CGP EST assembly CLS_S3_ESTs_Sat, EST library.pdf (EMS2) (PDF 40 kb) [file 11032_2016_606_MOESM2_ESM.pdf]

Table S2 Features of the CGP EST assembly CLS_S3_ESTs_Sat, EST library used to locate SNPs.

ASSEMBLY	SPECIES	GENOTYPE	LIBRARY	#_of_ESTs	CONTIGS	SINGLETS	UNIGENES	GROUPS	EST/UNI	UNI/GRP	LENGTH	COMMENTS
CLS_S3_Contig#	lettuce	*Lactuca sativa*	QG(ABCDI) CLS(XYZ) CLS(LMS)	76043	11455	17962	29417	22657	2.56	1.3	740	stringent cap3 conditions
