# Supplementary material for: Towards new sources of resistance to the currant-lettuce aphid (Nasonovia ribisnigri)
Source: Mol Breed. 2017 Jan 3;37(1):4. doi: 10.1007/s11032-016-0606-4 (PMC5209396; doi:10.1007/s11032-016-0606-4)
Supplement: Supplementary file 4 — Lactuca sativa Lsat_1_v4 pseudo-chromosome assembly.xlsx (ESM4) (PDF 24 kb) [file 11032_2016_606_MOESM4_ESM.pdf]

Table S4 Summary of the features in L. sativa genome assembly

Lsat_1_v4 from U. C. Davis (http://lgr.genomecenter.ucdavis.edu)

Pseudo-chromosome	No. LKAM SNPs	Marker spacing bp		
		Min	Mean	Max
1	69	197	3715404.838	55928185
2	92	135	2616368.733	42420715
3	28	85	9794877.296	51612469
4	103	170	3873700.725	75583322
5	81	54	5049854.038	47534847
6	42	137	5159484.049	48505829
7	82	458	2963070.679	33907280
8	67	481	4836685.523	38968829
9	22	8558	8968193.000	71692295
10	96	-	-	-
Total	682			
Total mapped	586			
Across all Chrs		54	5219737.654	75583322

accessed 24/09/12
