# Supplementary material for: Towards new sources of resistance to the currant-lettuce aphid (Nasonovia ribisnigri)
Source: Mol Breed. 2017 Jan 3;37(1):4. doi: 10.1007/s11032-016-0606-4 (PMC5209396; doi:10.1007/s11032-016-0606-4)
Supplement: Supplementary file 5 — Position of LKAMS in assembly.pdf (EMS5) (PDF 202 kb) [file 11032_2016_606_MOESM5_ESM.pdf]

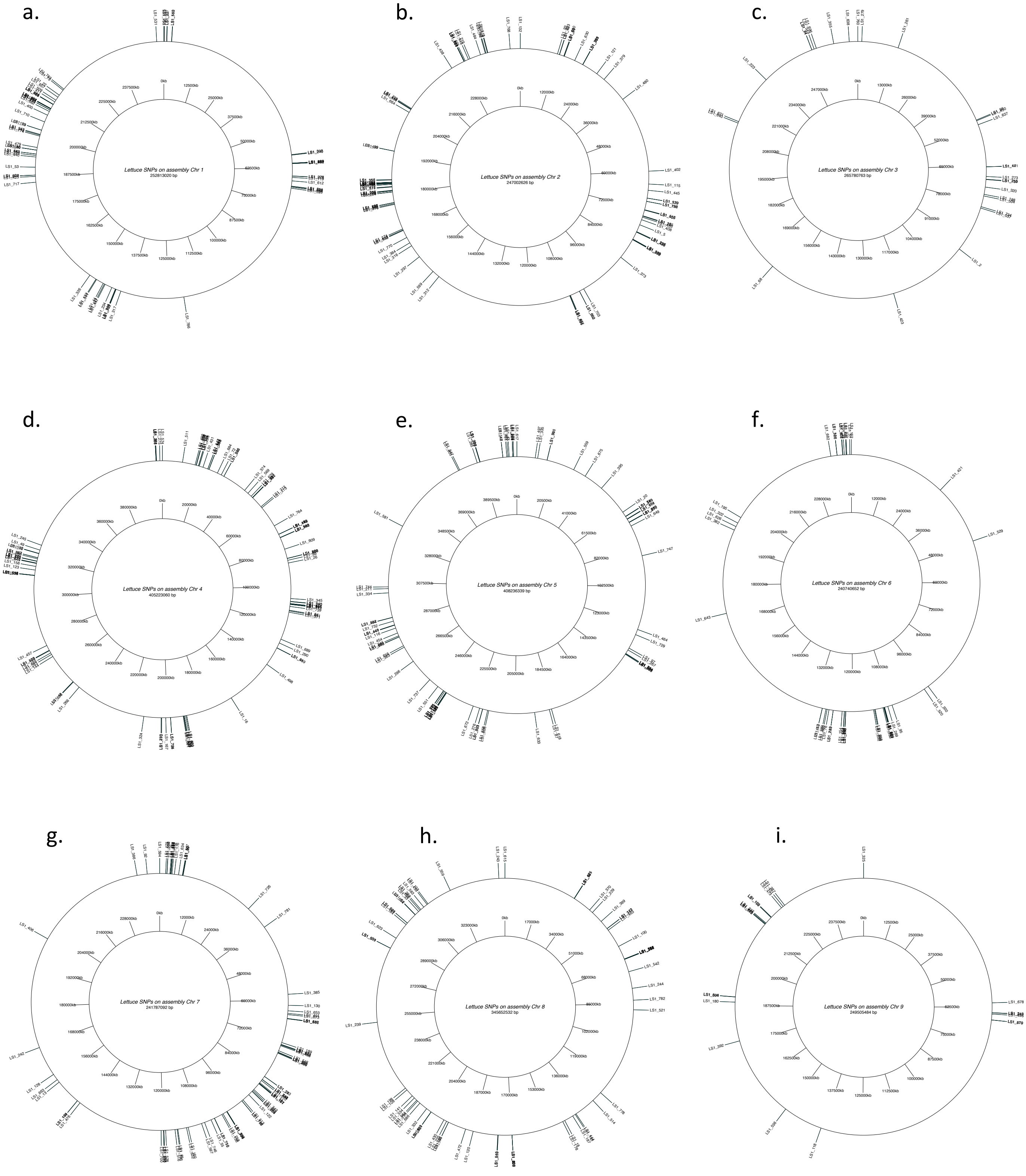


Figure S1 Relative positions of LKAM sequences in relation to the *L. sativa* draft genome assembly 'Lsat_1_v4'. a.- i., Pseudo-chromosomes 1 – 9 respectively. Circularized for illustration, the scale on the inner circle depicts base pair (bp) intervals, the outer circle is labeled with the LKAM markers prefixed LS1_ then the numeric identifier.
