# Supplementary material for: Towards new sources of resistance to the currant-lettuce aphid (Nasonovia ribisnigri)
Source: Mol Breed. 2017 Jan 3;37(1):4. doi: 10.1007/s11032-016-0606-4 (PMC5209396; doi:10.1007/s11032-016-0606-4)
Supplement: Supplementary file 6 — Heterozygozity and PIC of LKAMS.pdf (EMS6) (PDF 274 kb) [file 11032_2016_606_MOESM6_ESM.pdf]

a.

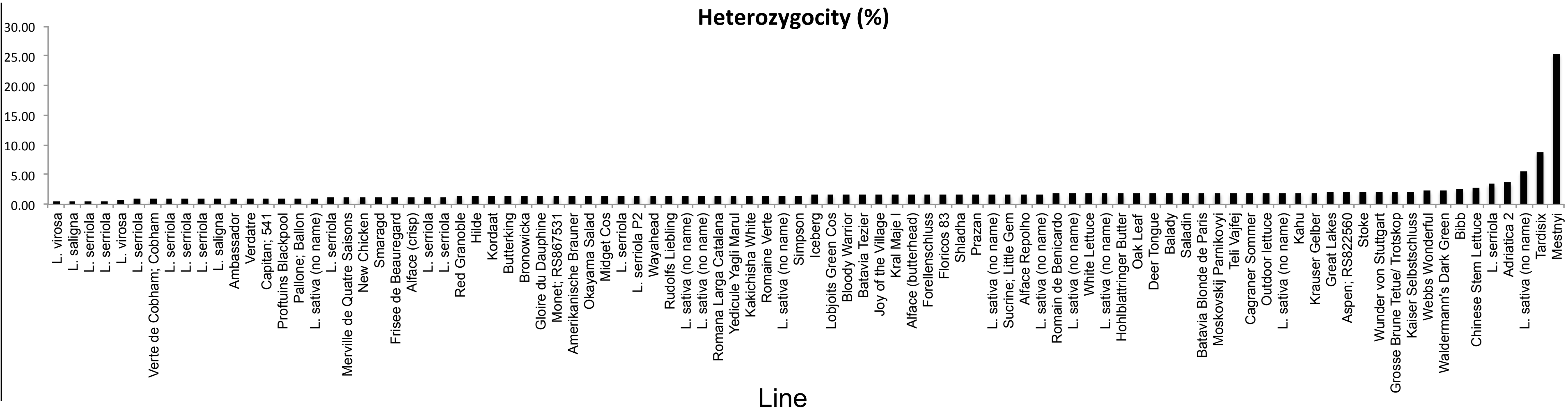

b.

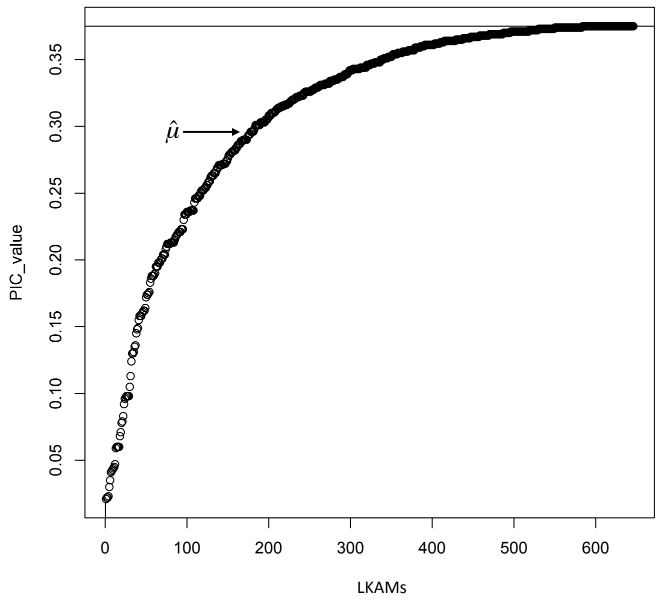

c.

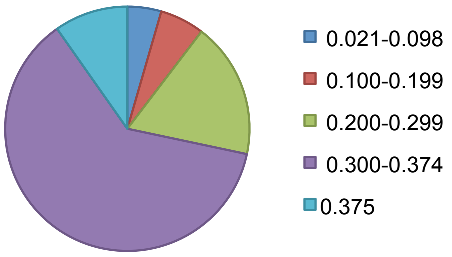

Supplementary Figure S2 LKAM genetic diversity:

a. Heterozygocity across the Lettuce diversity set
b. Distribution of PIC values
c. Proportions of PIC values
