# Supplementary material for: Towards new sources of resistance to the currant-lettuce aphid (Nasonovia ribisnigri)
Source: Mol Breed. 2017 Jan 3;37(1):4. doi: 10.1007/s11032-016-0606-4 (PMC5209396; doi:10.1007/s11032-016-0606-4)
Supplement: Supplementary file 7 — LD decay with distance.pdf (EMS7) (PDF 166 kb) [file 11032_2016_606_MOESM7_ESM.pdf]

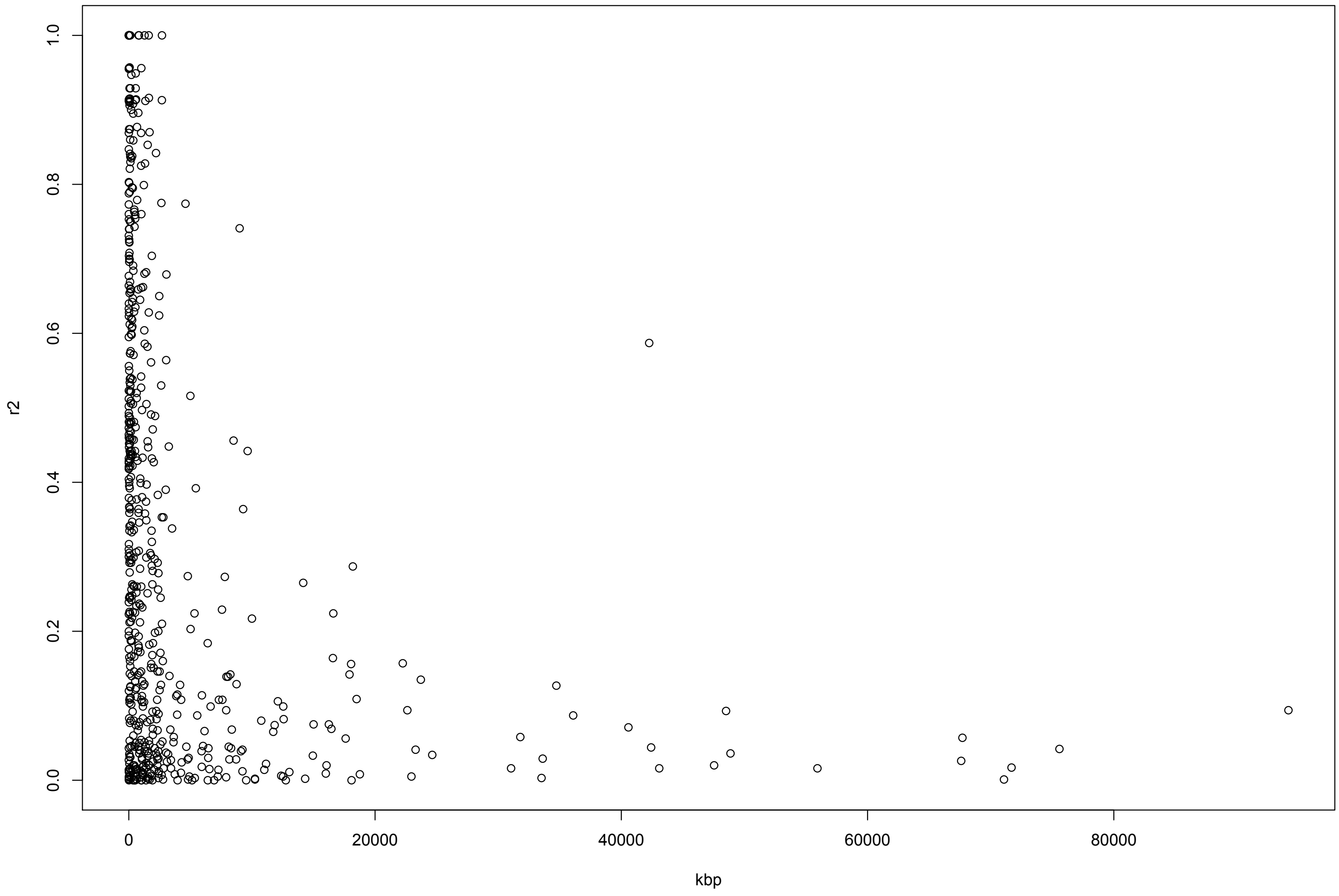

Figure S3 Plot of LD (in r^2) against weighted distance (kbp) between pairwise SNP comparisons across the L. sativa pseudo-chromosome assembly ‘Lsat-1_v4’ .
