# Supplementary material for: Towards new sources of resistance to the currant-lettuce aphid (Nasonovia ribisnigri)
Source: Mol Breed. 2017 Jan 3;37(1):4. doi: 10.1007/s11032-016-0606-4 (PMC5209396; doi:10.1007/s11032-016-0606-4)
Supplement: Supplementary file 8 — Pairwise rsq in assembly.pdf (EMS8) (PDF 381 kb) [file 11032_2016_606_MOESM8_ESM.pdf]

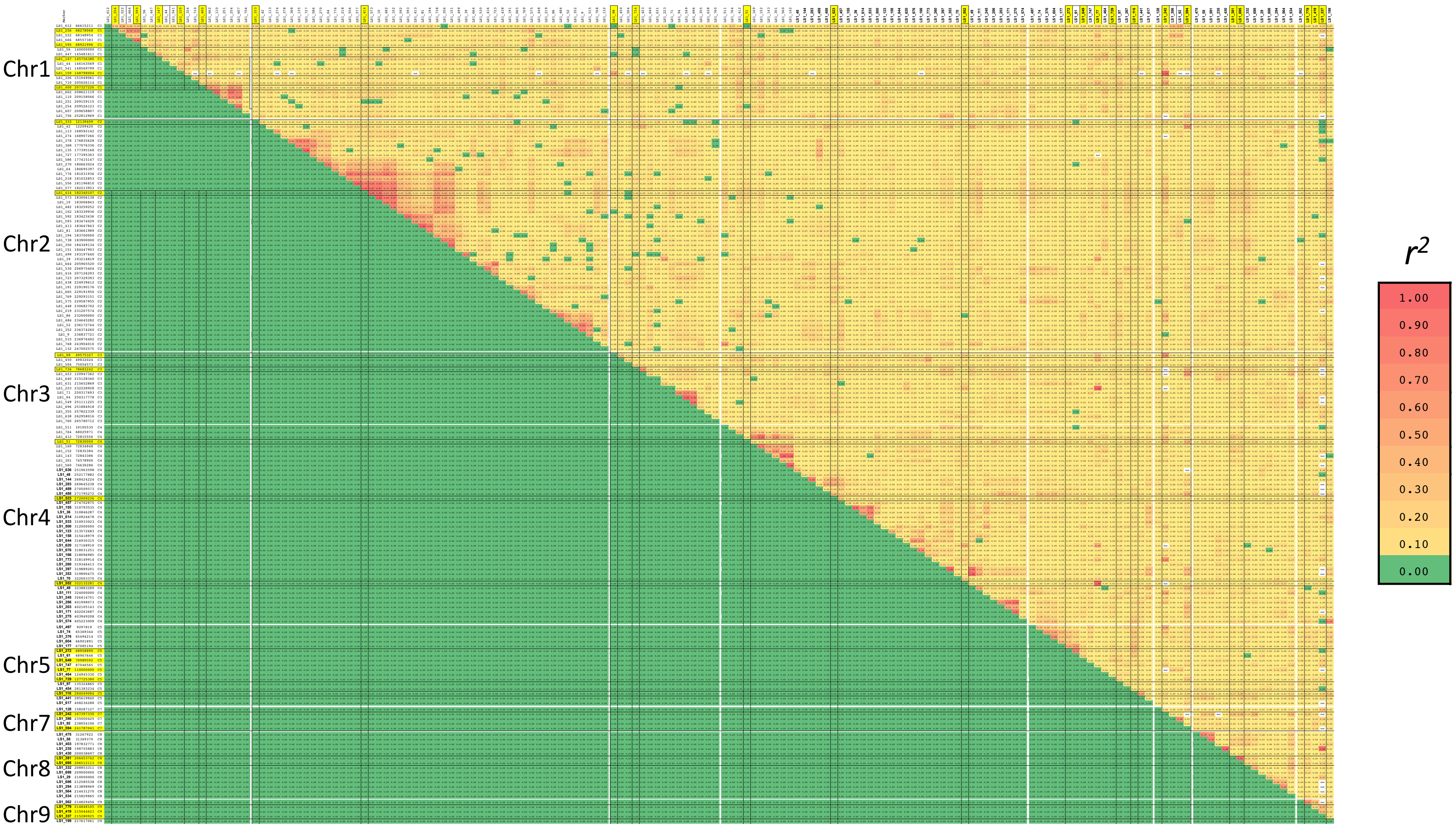


Figure S4 Pairwise |*r2* | for 167 SNPs across *L. sativa* pseudo-chromosomes 1, 2, 3, 4, 5, 7, 8, 9 from assembly 'Lsat-1_v4'. Yellow highlighting indicates SNPs associated with *Nasonovia* count data. The SNPs surrounding the significant SNPs are included for regional reference to the surrounding LD.
