# Supplementary material for: Towards new sources of resistance to the currant-lettuce aphid (Nasonovia ribisnigri)
Source: Mol Breed. 2017 Jan 3;37(1):4. doi: 10.1007/s11032-016-0606-4 (PMC5209396; doi:10.1007/s11032-016-0606-4)
Supplement: Supplementary file 9 — REML variance components.xlsx (EMS9) (PDF 30 kb) [file 11032_2016_606_MOESM9_ESM.pdf]

Table S5 REML variance components analyses for aphid count data from N. ribisnigri biotype Nr:0

REML variance components analysis for the numbers of Nr:0 alate (winged) aphids

Response variate:	SQRT((Winged+0.375))
Fixed model:	Constant + Name
Random model:	Rep + Rep.Block + Rep.Block.Plot
Number of units:	774 (70 units excluded due to zero weights or missing values)

Rep.Block.Plot used as residual term
Non-sparse algorithm with Fisher scoring

Estimated variance components

Random term	component	s.e.
Rep	1.473	0.762
Rep.Block	-0.056	0.084

Residual variance model

Term	Factor	Model(order)	Parameter	Estimate	s.e.
Rep.Block.Plot	Identity	Sigma2		4.699	0.27

Fixed term	Wald statistic	n.d.f.	F statistic	d.d.f.	F pr
Name	442.49	95	4.66	643.3	<0.001

minLSD	meanLSD	maxLSD
1.891	2.141	2.851

REML variance components analysis for number of Nr:0 apterous (non-winged) aphids

Response variate:	SQRT((Non_winged+0.375))
Fixed model:	Constant + Name
Random model:	Rep + Rep.Block + Rep.Block.Plot
Number of units:	774 (70 units excluded due to zero weights or missing values)

Rep.Block.Plot used as residual term

Non-sparse algorithm with Fisher scoring

Estimated variance components

Random term	component	s.e.
Rep	4.2	2.17
Rep.Block	-0.25	0.25

Residual variance model

Term	Factor	Model(order)	Parameter	Estimate	s.e.
Rep.Block.Plot	Identity	Sigma2		14.65	0.84

Tests for fixed effects

Sequentially adding terms to fixed model

Fixed term	Wald statistic	n.d.f.	F statistic	d.d.f.	F pr
Name	539.85	95	5.68	639.5	<0.001

Dropping individual terms from full fixed model

Fixed term	Wald statistic	n.d.f.	F statistic	d.d.f.	F pr
Name	539.85	95	5.68	639.5	<0.001

minLSD	meanLSD	maxLSD
3.327	3.769	5.025

REML variance components analysis for Nr:0 total aphid number

Response variate:	SQRT((Total+0.375))
Fixed model:	Constant + Name
Random model:	Rep + Rep.Block + Rep.Block.Plot
Number of units:	774 (70 units excluded due to zero weights or missing values)

Rep.Block.Plot used as residual term

Non-sparse algorithm with Fisher scoring

Estimated variance components

Random term	component	s.e.
Rep	5.22	2.7
Rep.Block	-0.25	0.29

Residual variance model

Term	Factor	Model(order)	Parameter	Estimate	s.e.
Rep.Block.Plot	Identity	Sigma2		16.83	0.97

Tests for fixed effects

Sequentially adding terms to fixed model

Fixed term	Wald statistic	n.d.f.	F statistic	d.d.f.	F pr
Name	581.46	95	6.12	641.4	<0.001

Dropping individual terms from full fixed model

Fixed term	Wald statistic	n.d.f.	F statistic	d.d.f.	F pr
Name	581.46	95	6.12	641.4	<0.001

minLSD	meanLSD	maxLSD
3.571	4.045	5.389
