# Supplementary material for: Towards new sources of resistance to the currant-lettuce aphid (Nasonovia ribisnigri)
Source: Mol Breed. 2017 Jan 3;37(1):4. doi: 10.1007/s11032-016-0606-4 (PMC5209396; doi:10.1007/s11032-016-0606-4)
Supplement: Supplementary file 11 — Manhattan and Q-Q plots with genomic control.pdf (EMS11) (PDF 219 kb) [file 11032_2016_606_MOESM11_ESM.pdf]

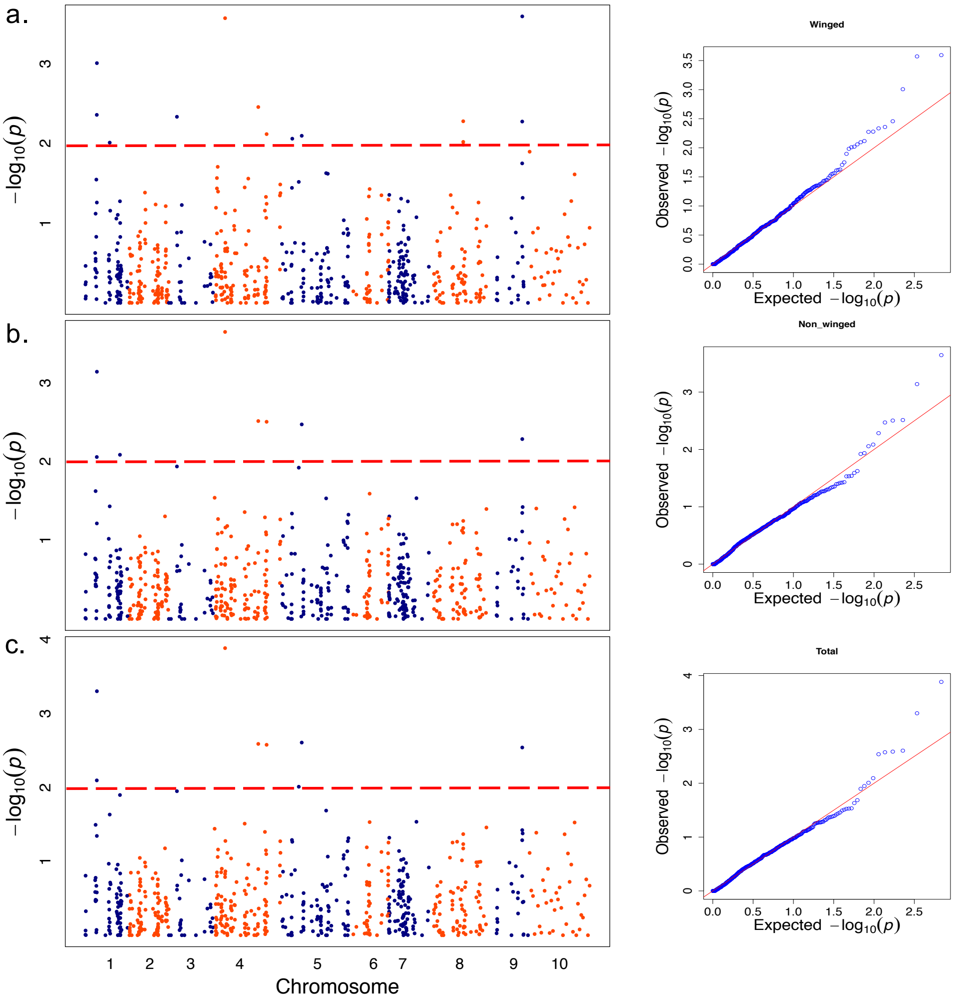

Figure S5 *Lactuca sativa* pseudo-chromosome distribution of –log10 *P*-values for 646 LKAM associations with *N. ribisnigri* count data obtained from the VeGIN lettuce diversity set. Panels' a., b., and c. represent SNP associations with mean count data for alate, apterous, total-aphid count respectively. Horizontal dashed line represents a threshold of –log10 *P*-value >2. Pseudo–chromosomes 1-9 and group-10 (un-mapped SNPs) alternately coloured blue and red. Each panel is accompanied with a Q-Q plot following genomic control for the three phenotype groups.
