# Supplementary material for: Towards new sources of resistance to the currant-lettuce aphid (Nasonovia ribisnigri)
Source: Mol Breed. 2017 Jan 3;37(1):4. doi: 10.1007/s11032-016-0606-4 (PMC5209396; doi:10.1007/s11032-016-0606-4)
Supplement: Supplementary file 14 — Putative roles of the LKAM SNPs significant in both association tests.xlsx (EMS14) (PDF 67 kb) [file 11032_2016_606_MOESM14_ESM.pdf]

Table S9 Putative role of the LKAM SNPs that were significant in both the Kruskal-Wallis and GWAS association tests.

Phenotype	LKAM (SNP)[a]	Alleles	*Arabidopsis* gene model	Description	Putative link to aphid resistance
Apterous	LS1_400	G/A	AT5G26880	tRNA/rRNA methyltransferase (SpoU) family protein	
Alate	LS1_98	**T**/C	AT2G25140	putative heat shock protein (HSP100)	These proteins not only protect against thermal damage but also have been shown to help protect against oxidative stress, caused by reactive oxygen species such as hydrogen peroxide. Lee et al. (2000) demonstrated that a chloroplast localized small heat shock protein was expressed following oxidative stress, in the absence of heat stress, they speculate that H_2O_2 itself can act to induce the expression of the heat shock protein.
Alate, apterous and total aphid	LS1_51	A/**C**	AT3G61540	Prolyl aminopeptidase (PAP)	Prolyl aminopeptidase (PAP) has been placed in the S3 family of serine peptidases, with the multimeric forms being found in plants (Kitazono et al., 1994; Szawłowska et al., 2011). These enzymes are capable of removing both proline and hydroxyproline from the N-terminus, and have been implicated in the regulation of free amino acid pools and the degradation of proteins damaged during oxidative stress (Walling and Gu, 1996). The related family of metallopeptidases, includes the leucine aminopeptidases (LAPs). This group of enzymes have been shown to be involved in plant defense against herbivory (Pautot et al., 1993). It may be that prolyl aminopeptidase participate in a signaling strategy through the release of smaller elicitor compounds from components in the aphid saliva, these could then be recognized elsewhere, indeed these elicitors may even up-regulate the protease.
Alate, apterous and total aphid	LS1_729	T/**C**	AT3G46290	HERCULES Receptor Kinase 1 (HERK1)	In *Arabidopsis* HERK1 is localized to the plasma membrane and forms part of a unique signaling pathway with two other CrRLK family RLKs, THE1 and FER, required for cell elongation during vegetative growth (Wolf et al., 2012). Indeed, *HERK1* has been shown to be up-regulated by gibberellic acid and induced by indole-3-acetic acid (Guo et al., 2009). Guo et al. (2009) proposed a pathway that has cross-talk, possibly via a common set of gene targets with the brassinosteroid signaling pathway, with *HERK1*, *THE1*, and *FER* being induced by brassinosteroids through BES1/ BZR1 transcription factors (regulated by BRI1 and BES2). Interestingly, the BRI1 receptor has been shown to bind BRI-1 associated kinase 1 (BAK1, also known as SERK3, somatic embryogenesis-related kinase-3) to mediate brasinosteroid signal transduction (Li et al., 2002; Nam and Li, 2002). BAK1 has been implicated in the regulation of plant defense (up-regulated by pathogen associated molecular patterns, playing a role in the reactive oxygen species burst response) and programmed cell death (Chinchilla et al., 2007; Heese et al., 2007; Kemmerling et al., 2007).
Alate	LS1_381	C/**T**	AT3G12750	Zinc transporter (ZIP1)	In plants, ZIP1 proteins have been suggested to play a role in the uptake of Zn from the rhizosphere (Grotz et al., 1998). Some plants accumulate Zinc as a means to deter herbivory (Persans and Salt, 2000; Pollard and Baker, 1997). The allelic variation at LS1_381 may be related to this process.
Alate	LS1_695	G/**A**	AT1G37130	Nitrate Reductase 2 (NR2)	Wilson et al. (2010) hypothesized that aphid herbivory (*Myzus persicae*) of brassicaceous plants leads to the up-regulation of nitrate reductase, possibly as a response to compounds present in aphid saliva. If this were the case then it maybe that certain allelic variants of *NR2* are preferential to the aphid, ensuring the host dose not become nitrogen depleted. Since NR2 is involved in the conversion of nitrate to nitrite, and that nitrite feeds into a series of reactions that lead to the formation of free glutamate which then makes up part of the amino acid profile injested by the aphids, it may be preferential to the aphid to select and colonise plants that they can influence *NR2* experession, with more resistant accession having an allelic variant that may not be as suitable.

[a]LKAM SNPs that have a significant association with the phenotypes alate, apterous, or total aphid number, determined by Kruskal-Wallis test and GWAS association. The allelic variant that is associated with a decrease in aphid number is underlined in bold-type.

Suplemenatary references for Table S9

Chinchilla, D., Zipfel, C., Robatzek, S., Kemmerling, B., Nürnberger, T., Jones, J., Felix, G., Boller, T. (2007) A flagellin-induced complex of the receptor FLS2 and BAK1 initiates plant defence. *Nature* , **448**, 497–500.

Grotz, N., Fox, T. C., Connolly, E., Park, W., Guerinot, M. L., and Eide, D. (1998) Identification of a family of zinc transporter genes from Arabidopsis that respond to zinc deficiency. *Proc. Natl. Acad. Sci. USA* , **95**, 7220–7224.

Guo, H., Li, L., Ye, H., Yu, X., Algreen, A., Yin, Y. (2009) Three related receptor-like kinases are required for optimal cell elongation in Arabidopsis thaliana. *Proc. Natl. Acad. Sci. USA* , **106**, 7648–53.

Heese, A., Hann, D. R., Gimenez-Ibanez, S., Jones, A. M., He, K., Li, J., Schroeder, J. I., Peck, S. C., Rathjen, J. P. (2007) The receptor-like kinase SERK3/BAK1 is a central regulator of innate immunity in plants. *Proc. Natl. Acad. Sci. USA* , **104**, 12217–12[illegible]

Kemmerling, B., et al. (2007) The BRI1-associated kinase 1, BAK1, has a brassinolide-independent role in plant cell-death control. *Curr. Biol* . **17**, 1116–1122.

Kitazono, A., Kitano, A., Tsuru, D., Yoshimoto, T. (1994) Isolation and characterization of the prolyl aminopeptidase gene (pap) from *Aeromonas sobria* : comparison with the Bacillus coagulans enzyme. *J. Biochem* . **116**, 818–825.

Lee, B. H., Won, S. H., Lee, H. S., Miyao, M., Chung, W. I., Kim, I. J., Jo, J. (2000) Expression of the chloroplast-localized small heat shock protein by oxidative stress in rice. *Gene* , **245**, 283–290.

Li, J., Wen, J., Lease, K. A., Doke, J. T., Tax, F. E., Walker, J. C. (2002) BAK1, an Arabidopsis LRR receptor-like protein kinase, interacts with BRI1 and modulates brassinosteroid signaling. Cell, **110**, 213– 222.

Nam, K. H., and Li, J. (2002) BRI1/BAK1, a receptor kinase pair mediating brassinosteroid signaling. *Cell* , **110**, 203–212.

Pautot, V., Holzet, F. M., Reisch, B., Walling, L. L. (1993) Leucine aminopeptidase: an inducible component of the defence response in *Lycopersicon esculentum* (tomato). *Proc. Natl. Acad. Sci. USA* , **90**, 9906-9910.

Persans, M. W., and Salt, D. E. (2000) Possible molecular mechanisms involved in nickel, zinc, and selenium hyperaccumulation in plants. *Biotechnol. Genet. Eng* . **17**, 389-413.

Pollard, A. J. and Baker, A. J. M. (1997) Deterrence of herbivory by zinc hyperaccumulation in *Thlaspi caerulescens* (Brassicaceae). *New Phytol* . **135**, 655–658.

Szawłowska, U., Zdunek-Zastocka, E., Bielawski, W. (2011) Biochemical characterisation of prolyl aminopeptidase from shoots of triticale seedlings and its activity changes in response to suboptimal growth conditions. *Plant Physiol. Bioch* . **49**, 1342-1349.

Walling, L. L., and Gu, Y. Q. (1996) Plant aminopeptidases: occurrence, function and characterization. In: A. Taylor (Ed.), Aminopeptidases. R.G. Landes Co, Austin, Texas

Wilson, A. C. C., Sternberg, L., Hurley, K. B. (2011) Aphids alter host-plant nitrogen isotope fractionation. *Proc. Natl. Acad. Sci* . **108**, 10220-10224.

Wolf, S., Hématy, K., Höfte, H. (2012) Growth control and cell wall signaling in plants. *Annu. Rev. Plant Biol* . **63**, 381-407.
